# Lentiviral vectors for hematopoietic stem cell gene therapy restore α-globin expression in α-thalassemia red blood cells

**DOI:** 10.1016/j.xcrm.2025.102362

**Published:** 2025-09-17

**Authors:** Eva E.R. Segura, Kevyn Hart, Beatriz Campo Fernandez, Devin Brown, Kevin Tam, Andrea Gutierrez Garcia, Eva Seigneurbieux, Karen Li, Carol Mulumba, Emma Blakely, Katelyn Masiuk, Roshani Sinha, Devesh Sharma, John Everett, Matthew Hogenauer, M. Kyle Cromer, Frederic Bushman, Tippi C. MacKenzie, Donald B. Kohn

**Affiliations:** 1 Molecular Biology Interdepartmental Doctoral Program, David Geffen School of Medicine at University of California, Los Angeles, Los Angeles, CA 90095, USA; 2 Department of Microbiology, Immunology & Molecular Genetics, David Geffen School of Medicine at University of California, Los Angeles, Los Angeles, CA 90095, USA; 3 Department of Human Genetics, David Geffen School of Medicine at University of California, Los Angeles, Los Angeles, CA 90095, USA; 4 Department of Biology at California State University, Northridge, Northridge, CA 91330, USA; 5 Department of Molecular, Cell, and Developmental Biology at University of California, Los Angeles, Los Angeles, CA 90095, USA; 6 Department of Surgery, Division of Pediatric Surgery at University of California, San Francisco, San Francisco, CA 94143, USA; 7 Department of Microbiology, Perelman School of Medicine at University of Pennsylvania, Philadelphia, PA 19104, USA; 8 Eli and Edythe Broad Center of Regenerative Medicine and Stem Cell Research at University of California, San Francisco, San Francisco, CA 94143, USA; 9 Center for Maternal-Fetal Precision Medicine at University of California, San Francisco, San Francisco, CA 94143, USA; 10 Department of Pediatrics (Hematology/Oncology), David Geffen School of Medicine at University of California, Los Angeles, Los Angeles, CA 90095, USA; 11 Eli and Edythe Broad Center of Regenerative Medicine and Stem Cell Research at University of California, Los Angeles, Los Angeles, CA 90095, USA

**Keywords:** alpha globin, lentiviral vectors, hemoglobin level, red blood cells, alpha thalassemia major, hematopoietic stem cell, gene therapy, autologous, anemia, beta-globin LCR

## Abstract

Alpha thalassemia major (ATM) is an inherited blood disorder caused by the absence of all four α-globin genes (*HBA2/1*), resulting in severe anemia and lifelong transfusion dependence. While allogeneic hematopoietic stem cell transplantation (HSCT) offers a potential cure, donor availability remains limited. We present a gene therapy approach for autologous HSCT using lentiviral vectors (LVs) to deliver *HBA2* under the regulation of optimized β-globin locus control region (LCR) enhancers, restoring α-globin expression in red blood cells. The best-performing LVs, erythroid vector-alpha (EV-α) and EV-α-UV, achieved up to 100% transduction efficiency in human hematopoietic stem and progenitor cells (HSPCs), optimal vector copy numbers, and safe integration profiles. ATM-derived HSPCs from three donors treated with these LVs yielded α/β-globin mRNA and chain ratios within the therapeutic range (∼0.5+), and restored hemoglobin levels by 50%–100%. These findings establish the safety and clinical potential of EV-α and EV-α-UV as a promising autologous stem cell gene therapy for ATM.

## Highlights

• β-globin regulatory elements drive the α-globin gene
• Vector restores α-globin expression in alpha thalassemia patient red blood cells
• Rebalance of α/β-globin ratio and functional hemoglobin production

## Graphical abstract

**Figure:**
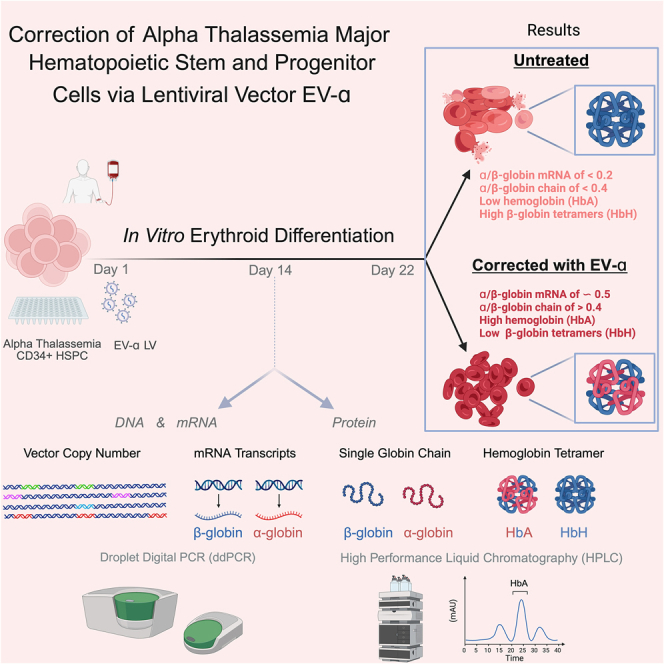

## Teaser

Segura et al. show that vectors encoding *HBA2* under β-globin regulatory elements restore α-globin expression in red blood cells derived from hematopoietic stem and progenitor cells of alpha thalassemia major (ATM) patients. Correction of the α/β-globin ratio enables hemoglobin production, supporting gene addition as a promising therapy for ATM.

## Introduction

Thalassemias are autosomal recessive hemoglobinopathies caused by mutations in the α-globin (*HBA2* and *HBA1*) or the β-globin (*HBB*) genes, which reduce production of the polypeptide chains that form adult hemoglobin (HbA), the molecule responsible for oxygen delivery in red blood cells (RBCs). *HBA2* and *HBA1* each encode identical α-globin chains, and their combined expression is required to maintain a balanced α/β-globin chain ratio. Reduced expression of α- or β-globin genes results in α- or β-thalassemia, respectively, with disease severity correlating to the number of defective alleles. Severe α-thalassemia, characterized by an α/β-globin chain ratio of <0.3 (normal ∼1.0), drastically limits HbA synthesis and causes abnormal erythroblast maturation, premature RBC precursor destruction, and long-term ineffective erythropoiesis, ultimately leading to anemia.^1^

The most prevalent forms of α-thalassemia are asymptomatic and result from the deficiency or absence of one to two α-globin genes, leading to a reduced α/β-globin chain ratio of ∼0.5.^2^ Hemoglobin H disease, caused by defects in three α-globin genes, presents with variable clinical severity and is characterized by the accumulation of unpaired β-globin chains, which form nonfunctional and toxic hemoglobin H (HbH) tetramers (β-globin tetramers). Alpha thalassemia major (ATM), the most severe form, arises from the deletion of all four α-globin genes and is typically lethal *in utero*. Advances in *in utero* transfusion (IUT) therapy, however, have demonstrated safety and positive postnatal neurological outcomes.^3^ For surviving individuals with severe disease, lifelong RBC transfusions combined with iron chelation therapy remain the standard of care, but iron toxicity continues to cause multi-organ complications.^4^

Allogeneic hematopoietic stem cell transplantation (HSCT) is the only curative treatment option for α-thalassemia. However, it is limited by donor availability and is accompanied by significant risks, including graft rejection, graft-versus-host disease (GVHD), and organ damage due to the high dosage of chemotherapy and immunosuppression required for engraftment.^5^^,^^6^ Gene therapy using autologous hematopoietic stem cells (HSCs) offers a promising alternative to allogeneic HSCT, circumventing donor-related limitations and eliminating GVHD risks. Clinical studies have demonstrated long-term and corrective outcomes for β-hemoglobinopathies using gene addition stem cell therapy with lentiviral vectors (LVs).^7^ These vectors are based on the human immunodeficiency virus 1 (HIV-1) but have been engineered to be replication-deficient and self-inactivating (SIN), ensuring safety for clinical use. Specifically, SIN LVs integrate a transcriptionally regulated β-globin gene into the genome of the patient's CD34+ hematopoietic stem and progenitor cells (HSPCs), enabling proper expression and hemoglobin produciton in RBCs. Such approaches have made remarkable progress in the treatment of β-thalassemia and sickle cell disease, with successful clinical trials and recent United States Food and Drug Administration approvals (e.g., Casgevy, Lyfgenia, and Zynteglo); however, α-thalassemia has historically received less attention, resulting in fewer therapeutic advancements.^8^

*In utero* RBC transfusions are currently used to support fetuses with ATM, improving survival and postnatal outcomes. Additionally, *in utero* allogeneic HSCT has been attempted as a curative approach, but these efforts have not resulted in long-term engraftment or immune tolerance. Building on these insights, our proposed approach is an *ex vivo*, postnatal gene therapy strategy where the fetus is maintained *in utero* via transfusions to mitigate disease-related morbidity, and definitive treatment would occur after birth. Specifically, HSPCs would be harvested from mobilized peripheral blood, transduced *ex vivo* with the lentiviral vector, and reinfused into the patient following myeloablative conditioning. Hence, we present the development of LVs for autologous HSC gene therapy targeting α-thalassemia. These α-globin erythroid vectors (α-globin EVs) are designed based on the β-globin LV, GLOBE, which has resulted in clinical success for treating β-thalassemia.^9^^,^^10^^,^^11^ Additional α-globin EVs were based on ultimate vector (UV), a shorter β-globin LV, developed in our laboratory with transcriptional regulatory elements of reduced length for optimized titer and CD34 infectivity.^12^ The α-globin EVs were engineered to deliver the *HBA2* gene under optimized transcriptional control, incorporating the β-globin promoter and the β-globin super-enhancers known as the β-globin locus control region (β-LCR).

This work evaluates α-globin EVs in CD34^+^ HSPCs derived from both healthy and α-thalassemia donors. Vector assessments include gene transfer efficiency, α-globin expression at both the mRNA and polypeptide levels, and hemoglobin production, thereby demonstrating their potential as a safe and effective gene therapy strategy for ATM.

## Results

### Design of α-globin EVs

**Figure 1:**
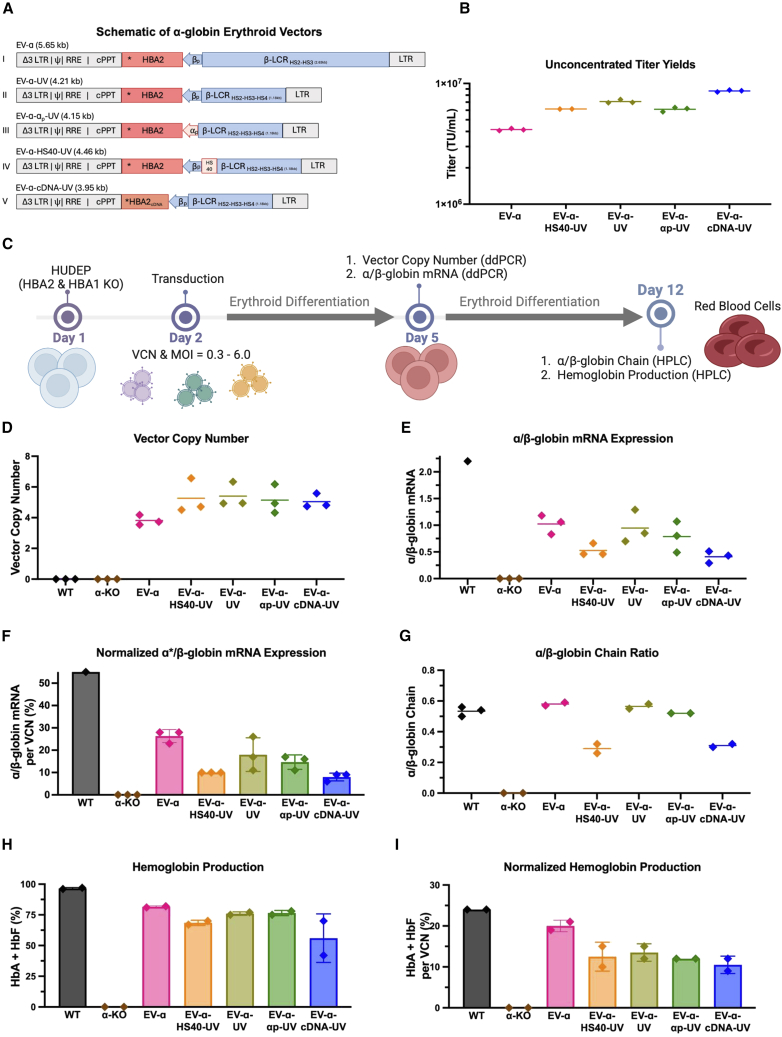
Design and assessment of ɑ-thal EVs in α-KO HUDEP-2 cells (A) Schematic representation of α-globin erythroid vectors (α-globin EVs) in their proviral forms. Viral elements are shown in gray: LTR, long terminal repeat with a 400 basepair (bp) deletion in the HIV U3 region (Δ3); packaging signal (φ or PSI), rev responsive element (RRE), central polypurine tract (cPPT). β-globin elements are in blue: β-globin core promoter (βp, 265 bp), β-globin locus control region (β-LCR) comprising combinations DNase I hypersensitive sites (HS2, HS3, and HS4; 1.2–2.6 kb). α-globin elements are shown in red: the *HBA2*∗ transgene (835 bp, including 3′ and 5′ UTRs and all introns) with a unique added 18 nucleotide sequence marker (TAG) for identification, the HS40 regulatory element (265 bp, also known as MSCR-2), and the α-globin core promoter (α_p_, 210 bp). The *HBA2* gene is in reverse orientation relative to vector transcription to prevent intron splicing. EV-α-cDNA-UV lacks introns. (B) Small-scale samples of each vector without concentration were produced in triplicates and their titer as determined in method details. (C) Experimental set-up: α-globin KO cells were transduced with α-globin EVs at 5E5 transducing unit (TU)/mL and cultured in erythroid differentiation conditions for 10 days, as described in the Method details. (D) Vector copy number (VCN) in α-KO HUDEP cells. (E) Gene expression analysis of transgene-derived α- and endogenous β-globin mRNA showing the α/β-globin ratio. (F) Gene expression normalized to corresponding VCN. (G) Ratio of α/β-globin chains in transduced α-KO HUDEP cells compared to the α/β-globin ratio in wild-type (WT) HUDEP cells. (H) Quantification of HbA and HbF normalized to all other peaks. (I) Normalized production of HbA and HbF to the corresponding VCN. Each data point represents a technical replicate, and error bars represent the mean with standard deviation.

Erythroid-specific α-globin EVs were designed by incorporating the α-globin gene (*HBA2*) along with various combinations of α- and/or β-globin regulatory elements into an SIN LV backbone (Figure 1A). Only short-to medium-sized LVs with proviral lengths ranging from 3.95 to 5.65 kilobase pair (kb) were designed to preserve high titer yields and gene transfer efficiency.^12^^,^^13^^,^^14^ The DNase I hypersensitive sites (HSs) from the β-LCR were integrated in every α-globin EV to retain erythroid-specific transgene expression.

Each α-globin EV series was categorized based on the size of their β-LCR element. The first group consisted of medium-sized vectors, including erythroid vector α-globin (EV-α), which integrates the 2.6 kb β-LCR region (HS2 and HS3) derived from the GLOBE vector (Figures 1A-I).^9^ The second group consisted of short-sized vectors, such as EV-α-UV, all harboring the 1.2 kb β-LCR region (HS2, HS3, and HS4) from the UV vector (Figures 1A-II). Both parental vectors, EV-α and EV-α-UV, share the core β-globin promoter and lack α-globin regulatory elements.

To examine the impact of α-globin regulatory elements, additional constructs were developed based on these parental LVs. EV-α-α_p_ from the GLOBE series (Figure S1A-I) and EV-α-α_p_-UV from the UV series (Figures 1A-III) include the endogenous 210 bp α-globin promoter core. Variants with extended α-globin promoter regions (450 and 750 bp) were also assessed in UV-based vectors (Figure S1A–II-III). The inclusion of the HS40 α-globin enhancer was also evaluated in EV-α-HS40-UV (Figures 1A-IV).^15^ Additionally, modifications to the *HBA2* gene were explored; EV-α-cDNA-UV incorporates the *HBA2* gene without introns (Figures 1A-V), and EV-α-ΔIVS2 (Figure S1A–IV) lacks intron 2. Lastly, EV-α-HBB/A-UV replaces the endogenous UTRs of *HBA2* with those of the β-globin gene (Figure S1A-V).

All α-globin EVs were packaged in HEK 293T cells with a disrupted *PKR* gene.^16^ Each α-globin EV yielded high titers and showed an inverse correlation with vector proviral length (Figure 1B). Raw vector supernatants were collected for functional titering and assays in a cell line, and concentrated viruses were used for primary cell assays.^16^

### Assessment in α-globin KO HUDEP-2 cells

α-globin EVs were assessed in the human umbilical cord blood-derived erythroid progenitor (HUDEP-2) cell line, which can undergo erythroid differentiation into HbA-producing RBC-like cells.^17^ These HUDEP-2 cells were further gene edited into an α-globin knockout (KO) cell line with a homozygous 20 kb deletion encompassing both *HBA2/1* genes (a-KO HUDEP-2). The modification eliminates endogenous α-globin expression, allowing for exclusive transgene-derived *HBA2* expression upon transduction.

Transduction with α-globin EVs, followed by a 10-day erythroid differentiation, was performed to assess transgene-derived α-globin mRNA and α-globin chain expression (Figure 1C). Hemoglobin tetramers were also analyzed to confirm proper assembly of the α-globin transgene chain with the β-globin chain. Analysis in differentiated cells revealed successful transduction and α-globin transcription across all α-globin EVs, as measured by vector copy number (VCN) and α-globin to β-globin mRNA ratio (α/β-globin), respectively (Figures 1D–1E). The normalization of mRNA expression with VCN revealed that EV-α was the highest expressing vector, with expression level reaching 30% per VCN, while EV-α-HS40-UV, despite harboring both enhancers, was one of the lowest expressing vectors (Figure 1F). While EV-α-cDNA-UV yielded a 10% decrease in transgene expression compared to other UV-based vectors retaining the full *HBA2* gene, no marked difference in transgene expression was observed in EV-α-ΔIVS2 (Figure S1B). No increase in *HBA2* transgene expression was observed when regulated by either the core *HBB* or *HBA2* promoters, nor with extended α-globin promoter length (Figures S1C–S1E). A decrease in gene regulation and expression was observed with the removal of endogenous *HBA2* UTRs, assessed in EV-α-HBB/A-UV (Figure S1B).

High-performance liquid chromatography (HPLC) analysis demonstrated that EV-α produced the most α-globin chains, reaching the expression level of those of wild-type (WT) HUDEP-2, as evaluated by the α/β-globin chain ratio (Figure 1G). Despite a high VCN, EV-α-cDNA-UV and EV-α-HS40-UV produced low levels of α-globin chains. The well-documented phenomenon in α-thalassemia patient cells in which the absence of α-globin genes leads to an upregulation of both γ-globin genes was observed in the α-KO HUDEP-2 cell line, but was effectively and proportionally corrected with increased transgene expression (Figure S2A).^18^ The absence of α-globin led to the formation of β-globin dimers, a phenotype also observed in the α-KO HUDEP-2 cell line and reversed with increased transgene expression (Figures S2B and S2C).

Additional HPLC analysis confirmed that the α-globin chains produced from the *HBA2* transgene properly assembled with the β- and γ-globin chains to form HbA and the fetal hemoglobin (HbF), respectively (Figure S2D). Most α-globin EVs achieved up to ∼75% hemoglobin reconstitution (Figure 1H). All α-globin EVs resulted in 10%–20% hemoglobin reconstitution per copy number, with EV-α yielding the highest hemoglobin production (Figure 1I). Differentiated RBCs, originally transduced with EV-α or EV-α-UV, were predominantly reconstituted with HbA with a low prevalence of HbH, closely resembling the hemoglobin profile of WT HUDEP-2 RBCs. In contrast, HbH remained the major hemoglobin peak in the EV-α-HS40-UV and EV-α-cDNA-UV samples (Figures S2D and S2E). Overall, analysis demonstrated that all α-globin EVs were functional in a human erythroid cell line, prompting a more extensive vector characterization of parental vectors, EV-α and EV-α-UV, in the α-globin KO HUDEP-2 cell line.

### Further vector characterization in α-KO HUDEP-2 cells

**Figure 2:**
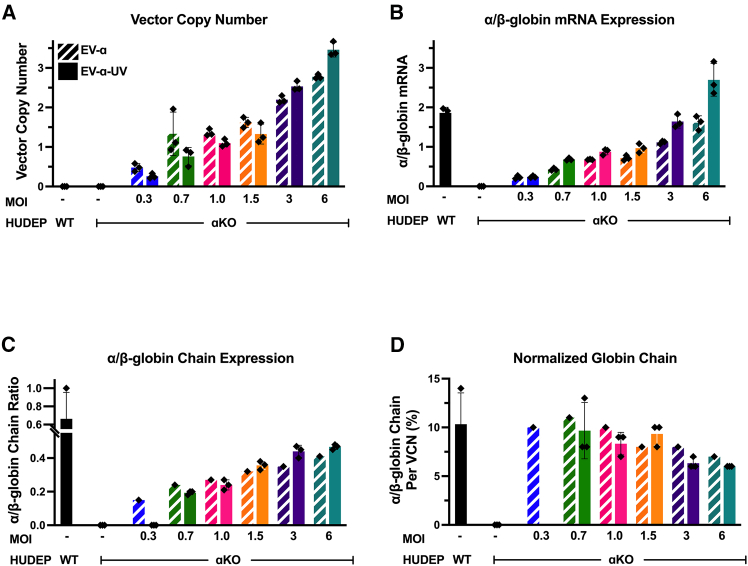
Dose-response assessments of α-globin EVs in α-KO HUDEP-2 cells α-KO HUDEP cells were transduced with EV-α and EV-α-UV at varying multiplicities of infections (MOI) (0.3–6), corresponding to virus concentration ranging from 7E4 to 1E6 TU/mL. (A) VCN analysis. (B) α/β-globin mRNA ratio in transduced α-KO HUDEP cells compared to α/β-globin ratio in WT cells. (C) Quantification of α/β-globin chains (α/β-globin for WT cells) by single globin chain HPLC. (D) Normalized α/β-globin chain expression per *HBA* copy, or VCN, or by endogenous copies (4) in WT HUDEP cells. Each data point represents a technical replicate and error bars represent mean with standard deviation.

Extensive characterization was performed on EV-α and EV-α-UV to assess gene transfer and gene expression across varying vector concentrations and multiplicities of infection (MOIs). Evaluation of VCNs demonstrated a dose-dependent gene transfer efficiency (Figure 2A). Correspondingly, *HBA2* transgene levels exhibited dose- and VCN-dependent expression, resulting in α/β-globin mRNA ratios ranging from 0.2 to 1.8, reaching the ratio of WT HUDEP-2 cells (Figure 2B).

VCN-dependent α-globin chain expression was also observed, resulting in an α/β-chain ratio ∼0.47 (with an MOI of 6 and VCN of 2), reaching that of WT HUDEP-2 cells of 0.66, which is equivalent to ∼71% of WT HUDEP-2 levels (Figure 2C). The α-globin chain expression per EV-α copy reached similar expression levels to that of one endogenous α-globin gene (Figure 2D).

### Characterization in healthy donor CD34^+^ cells

To achieve a more clinically relevant assessment, concentrated vectors were evaluated in healthy human primary bone marrow (BM)-derived CD34^+^ HSPCs. An 18-nucleotide transcriptional tag (TAG) was integrated downstream of the STOP codon to distinguish transgene-derived α-globin mRNA (α∗) from endogenous *HBA2/1* transcripts, allowing for precise quantification of α∗/β-globin mRNA using specific primer/probe sets. Prior to assessment in primary cells, these newly modified vectors were examined in α-KO HUDEP-2 cells and demonstrated a comparable raw and concentrated titer, gene transfer efficiency, and expression levels to their unmodified counterparts, without affecting α-globin production or hemoglobin assembly (Figures S3A–S3D).

**Figure 3:**
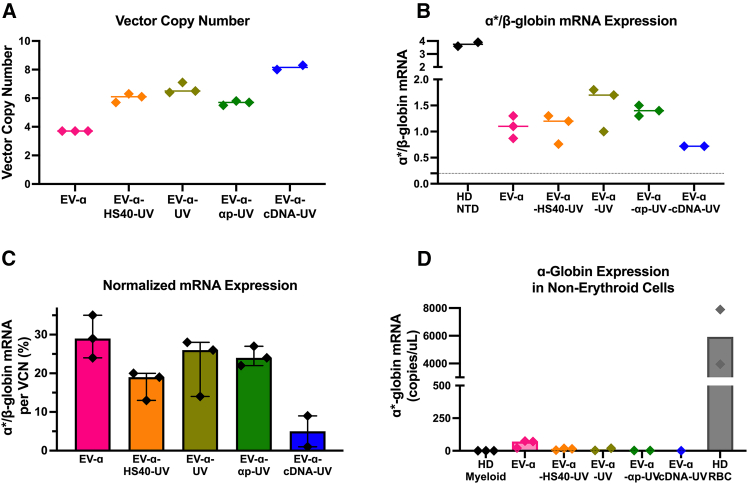
α-Globin transgene expression in differentiated erythroid cells derived from healthy donor (HD) CD34^+^ HSPCs CD34^+^ HSCPs from three healthy donors (HD) were transduced with the α-globin EVs at 2E7 TU/mL and cultured for 12 days under erythroid differentiation conditions and analyzed on day 14. (A) VCN analysis. (B) Gene expression analysis of transgene-derived α∗-globin mRNA and endogenous β-globin mRNA to determine α∗/β-globin ratio. (C) α∗/β-globin mRNA ratio normalized to the corresponding VCN. Following transduction, some cells were cultured in myeloid conditions to assess off-lineage transgene expression. (D) Assessment of α∗-globin mRNA expression in short-term myeloid cells cultured under myeloid conditions. Each data point represents a technical replicate, and error bars represent median, and median with 95% confidence interval in (C).

CD34^+^ HSPCs were pre-stimulated, transduced (with concentrated vectors), and cultured under erythroid differentiation conditions for 12 days, after which VCN and mRNA expression were assessed. As expected, VCNs inversely correlated with vector length, consistent with observations from β-globin LV series (Figure 3A).^12^ While maintaining a VCN ∼5, the absolute ratio of α∗-globin transgene mRNA to endogenous β-globin mRNA ranged from 0.7 to 1.8 across all α-globin EVs (Figure 3B). These findings confirmed that the α-globin transgene can be regulated by β-globin elements in differentiated primary human erythroid cells.

All α-globin EVs harboring the full *HBA2* gene achieved 20%–30% α-globin mRNA expression per VCN (Figure 3C). EV-α produced the highest α-globin gene expression per VCN of ∼30%, whereas EV-α-cDNA-UV, which lacks both introns, resulted in a 6-fold reduction, reaching ∼5% per VCN. Notably, no detectable transgene-derived α-globin expression was observed in myeloid cells (Figure 3D).

EV-α from the GLOBE series was selected as the primary candidate LV due to its highest α-globin expression yields. Although EV-α-UV and EV-α-α_p_-UV exhibited similar performance, EV-α-UV slightly outperformed in titer yields, gene transfer efficiency, and expression levels, making it the lead vector in the UV series. Codon optimization—applied to the entire transgene or limited to the first exon—evaluated in EV-α-UV did not enhance expression (Figures S4A–S4C).

### Characterization in CD34^+^ cells from human bone marrow and mobilized peripheral blood

**Figure 4:**
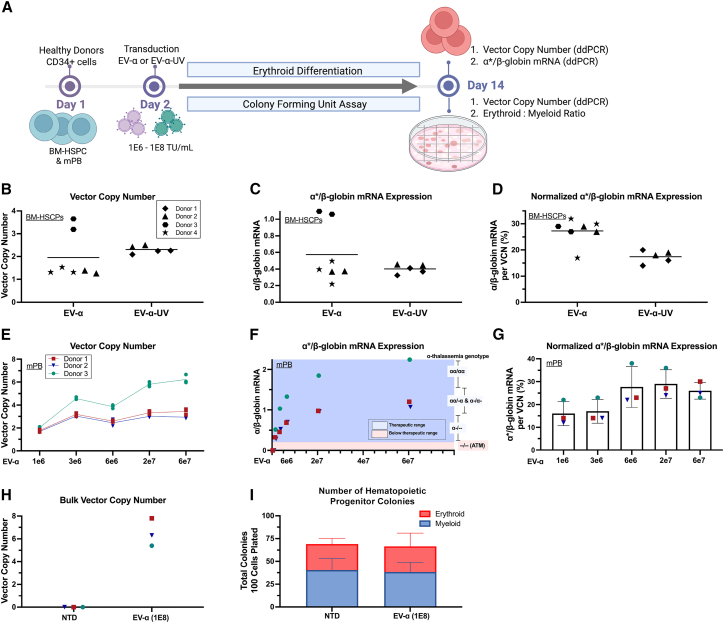
Evaluation of candidate vector potency and toxicity in CD34^+^ HSPCs from human bone marrow (BM) and mobilized peripheral blood (mPB) (A) Experimental setup for transduction and erythroid differentiation. BM-derived CD34^+^ from four HDs were transduced with EV-α and EV-α-UV vectors and analyzed on day 14. (B–D) BM-derived CD34^+^ cells were analyzed for (B) VCN, (C) α∗/β-globin ratio, and (D) α∗/β-globin ratio normalized by VCN. (E–G) mPB stem cells (mPBSCs) from three HDs were transduced with increasing doses of EV-α (1E6 to 6E7 TU/mL; MOIs: 2, 6, 13, 45, and 150) using 1 mg/mL Lentiboost. Analysis includes (E) VCN, (F) α∗/β-globin mRNA ratio, and (G) normalized gene expression. Shaded blue and red regions indicate α/β-globin mRNA threshold for blood-transfusion-free and transfusion-dependent individuals (<0.2), respectively. The ranges of α/β-globin mRNA ratios expected in patients with different α-thalassemia genotypes are shown on the right axis. (H and I) To assess potential cytotoxicity from the EV-α-LV at high VCN, CD34^+^ mPBSC from additional HDs were transduced at 1E8 TU/mL, and cells were analyzed for (H) VCN in short-term myeloid cells and, (I) CFU assay to quantify erythroid (red) and myeloid (blue) colonies produced in non-transduced (NTD) and high dose EV-α transduced cells (EV-α). Each symbol in (B–D) represents an independent biological replicate (different donors); repeated symbols for the same donor indicate technical replicates. In (E–G), different colors represent different donors, and each dot indicates a technical replicate. Error bars represent mean and mean with standard deviation.

The potency of EV-α and EV-α-UV was further evaluated in healthy donor (HD) CD34^+^ HSPCs (Figure 4A). At an equal transduction concentration of 2E7 transducing units (TU)/mL, both vectors achieved comparable VCNs and transgene expression levels, yielding α∗/β-globin mRNA of ∼0.45 (Figures 4B and 4C). When normalized per VCN, EV-α achieved 27% of α-globin expression, whereas EV-α-UV resulted in 17% (Figure 4D).

Characterization of EV-α in the intended clinical starting cell source, mobilized peripheral blood (mPB) CD34^+^, demonstrated a dose-dependent VCN (Figure 4E). The resulting α∗/β-globin mRNA ratios ranged between 0.3 and 1.8, which achieved the therapeutic range of individuals with asymptomatic α-thalassemia and of healthy individuals (Figure 4F). This corresponded to 15%–30% α-globin expression per integrated *HBA2* copy (Figure 4G).

Cytotoxicity was examined by transducing EV-α at a viral concentration of 1E8 TU/mL (5-fold higher than the intended clinical dose). Cells were then plated in methylcellulose medium and cultured under myeloid conditions for 14 days. Resulting HSPC colonies had a bulk VCN ranging from 6 to 8, and showed no decrease in clonogenicity or lineage skewing, as demonstrated by 70% of colony growth and a 1.3 myeloid-to-erythroid ratio in both transduced and non-transduced conditions (Figures 4H and 4I).

### Assessment of HSPCs engrafted in NOD/SCID/gamma (NSG) mice

**Figure 5:**
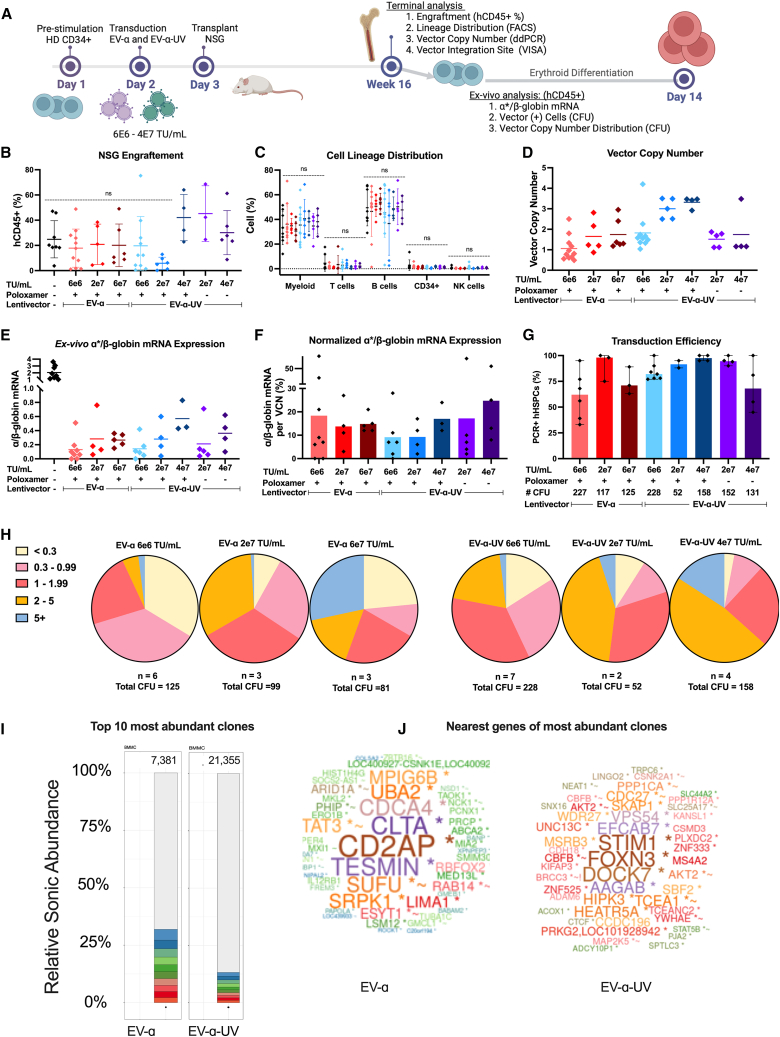
Long-term assessment of transduced HSPCs engrafted in NSG mice (A) Healthy donor CD34^+^ mPBSCs were transduced with EV-α and EV-α-UV at low (6E6 TU/mL, MOI: 6), medium (2E7 TU/mL, MOI: 20), and high (4E7 or 6E7 TU/mL, MOIs: 20 and 32, respectively) doses and transplanted into NSG mice. At 16 weeks post-transplant, mice were euthanized, and bone marrow (BM) hCD45^+^ cells were analyzed. Erythroid differentiation of BM hCD45^+^ was performed *ex vivo* to assess α-globin transgene expression. To evaluate VCN distribution, a colony forming unit assay using methylcellulose cultures was conducted using BM hCD45^+^. (B–D) BM analysis for (B) engraftment (percentage of hCD45^+^ cells out of total [human + mouse] CD45^+^ cells), (C) lineage distribution, and (D) bulk VCN of engrafted cells. (E and F) *Ex vivo* RBC differentiation for (E) α∗/β-globin mRNA ratio and (F) normalized gene expression to VCN. (G and H) Transduction profiles of engraftment HSPCs using CFU assay, showing (G) percentage of VCN+ colonies and (H) VCN values in single colonies. (I) Vector integration site analysis (VISA), performed by ligation-mediated PCR and next generation-sequencing, and aligned to the human genome, showing the relative abundance of the top 10 most abundant clones, shown in different colors. Lower abundance clones are shown in gray. Total number of unique integrants shown on top of each bar. (J) Word cloud summarizing nearest genes to integration sites, indicating transcription units (∗), within 50 kb of a cancer-related gene (∼), and the nearest gene associated with lymphoma (!). Bars represent mean, and error bars represent mean with standard deviation. Analysis of NSG assay was based on ANOVA (analysis of variance) across all arms with selected statistics shown, with *p* < 0.05 considered significant. Non-significance (ns) was set at *p* < 0.05. Each data point represents an individual biological replicate (one mouse).

Vector efficiencies of EV-α- and EV-α-UV-modified human HD HSPCs were evaluated in murine xenografts. CD34^+^ mPB cells were transduced with concentrated vectors using LentiBOOST at varying vector concentrations (6E6, 2E7, and 4E7 or 6E7 TU/mL) and then transplanted into sub-lethally irradiated NSG mice via retro-orbital injection (Figure 5A). The EV-α-UV groups were also subjected to transduction without LentiBOOST to evaluate its effects on transduction and gene transfer efficiency. At 16 weeks post-transplant, human cell engraftment and lineage contributions were analyzed, with no significant differences observed (Figures 5B and 5C). The bulk VCN in human cells from mouse BM ranged from 0.49 to 2.95 for EV-α-transduced cells and 1.04 to 4.20 for EV-α-UV-transduced cells (Figure 5D).

The α-globin transgene expression levels were measured *ex vivo*, as the NSG xenograft model does not support *in vivo* human erythropoiesis. RBCs differentiated from the engrafted human CD45^+^ cells yielded an α∗/β-globin ratio exceeding 0.20, with some ratios reaching as high as 0.83 (Figure 5E). While long-term *ex vivo* expression per VCN showed variability, EV-α averaged ∼20%, with similar levels observed for EV-α-UV at 4E7 TU/mL (Figure 5F).

The percentage of vector-positive progenitors (VCN > 0.3) and VCN in individual colonies were evaluated from human HSPCs explanted from NSG mice via a methylcellulose plating assay. Optimal transduction profiles of 100% vector-positive cells were achieved at concentrations of 2E7 and 4E7 TU/mL (MOIs of 32 and 20) for EV-α and EV-α-UV, respectively (Figure 5G). Analysis of individual transduced HSPC colonies revealed a narrow VCN range within the optimal clinical range (below 5), with over 95% of vector-positive myeloid colonies showing a VCN between 0.3 and 5 at LV doses of 6E6 and 2E7 TU/mL for both vectors (Figure 5H).

The distribution and clonal abundance of vector integration sites in engrafted human BM CD45^+^ cells using six mice per vector were analyzed via ligation-mediated PCR.^19^^,^^20^ Vector integration analysis demonstrated a polyclonal diversity pattern, as evidenced by the low relative abundance (<20%) of the 10 most abundant clones in each sample, and high values of the Shannon index (Figure 5I; Table S1). Notably, no integration sites were found in or near proto-oncogenes, such as *LMO2* and *MECOM*, which are associated with expanded clones (Figure 5J; Table S1). Both vectors showed a decrease in the number of unique integrants and overall polyclonal diversity *in vivo* compared to pre-transplanted cells, particularly in samples with low human cell engraftment (Figures S5A and S5B). To further assess genomic safety, we compared integration site distributions of EV-α and EV-α-UV to those from lentiviral vector-based clinical trials targeting cancer (CART19 T cells), chronic granulomatous disease (CGD), and β-thalassemia, all of which reported no integration-mediated adverse events. Each dataset included pre-infusion and post-infusion samples, and integration patterns were analyzed using receiver operating characteristics (ROC) area-based enrichment analysis across multiple genomic features and window sizes. While mild enrichment near gene bodies and repetitive elements was observed—consistent with known lentiviral vector preferences—EV-α and EV-α-UV vectors showed no enrichment near high-risk regions such as transcription start sites, CpG islands, or COSMIC oncogenes. Overall, the integration profiles of EV-α and EV-α-UV closely mirrored those of clinically validated vectors, supporting a favorable genomic safety profile (Figure S5C).

### Restoration of α-globin expression in cells from three patients with ATM

**Figure 6:**
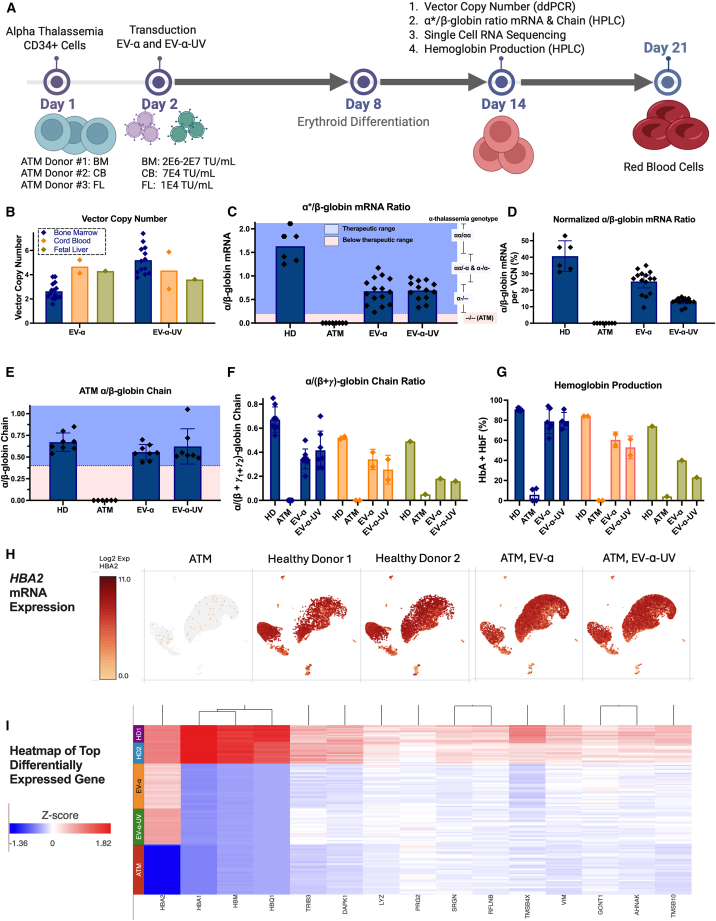
Restoration of α-globin expression in differentiated erythroid cells derived from CD34^+^ HSPCs from three patients with ATM (A) Experimental setup for transduction, with EV-α or EV-α-UV, and differentiation of bone marrow (BM), cord-blood (CB), and fetal liver (FL) CD34^+^ HSPCs. BM cells were transduced at doses ranging from 2E6 to 2E7 TU/mL, CB at 7E4 TU/mL, and FL at 1E4 TU/mL, then analyzed for vector copy number (VCN), mRNA expression, and protein production via HPLC on days 14 and 21 (BM: blue, CB: orange, FL: green). (B) VCN analysis. (C) α/β-globin mRNA ratio. (D) Normalized mRNA expression. Shaded regions represent the threshold for transfusion-free (blue) and transfusion-dependent (red) individuals (α/β-globin mRNA ratio <0.2). The ranges of α/β-globin mRNA ratios expected in patients with different α-thalassemia genotypes are shown on the right axis. (E) α/β-globin chain ratio in BM cells. (F) α/(β + γ_1_ + γ_2_)-globin chain ratio. (G) HbA and HbF production as a percentage of total HPLC peaks. (H and I) BM cells analyzed from single-cell RNA-seq. (H) UMAP showing *HBA2* mRNA expression and distribution in *in vitro* differentiated erythroid cells for each condition. Each plot displays levels of *HBA2* expression, indicated by the color scale to the right of each figure. (I) Heatmap of top 15 most differentially expressed genes. Each data point in (B–G) represents a replicate measurement on BM, CB, and FL CD34^+^ HSPCs. For BM samples (blue), data include technical replicates as well as independent experiments performed at different transduction doses using cells from the same donor. Colors indicate donor source. Bars represent mean with standard deviation. See Table 1 for detailed α/β-globin ratios.

The efficacy of EV-α and EV-α-UV was assessed in CD34^+^ cells from ATM donors harboring the 20 kb Southeast Asian (SEA) deletion, obtained from three cell sources: one from BM, one from cord blood (CB), and one fetal liver (FL) (Table S2; Figure 6A). Transduction conditions were adjusted for each cell source, reflecting the higher transducibility of more primitive cells. VCNs ranged from 1.58 to 5.44 for EV-α and from 2.82 to 7.40 for EV-α-UV (Figure 6B). The α/β-globin mRNA ratio was assessed for BM samples only, as RBCs from CB- and FL-derived CD34^+^ cell sources expressed γ-globin. BM-derived RBCs showed a similar mean α/β-globin mRNA ratio of 0.67, with EV-α ratios ranging from 0.23 to 1.17 and EV-α-UV ranging from 0.33 to 0.97. All samples ranged within the therapeutic range, reflecting ratios observed in asymptomatic patients and in healthy donors (Figure 6C; Table 1). EV-α achieved therapeutic α/β-globin ratios (>0.5) at VCNs as low as 1.5, indicating high expression efficiency even at low integration levels. Notably, at VCNs around 4, EV-α restored α/β-globin ratios approaching 1.0—comparable to those observed in healthy donors—suggesting that each integrated copy may drive expression at levels similar to an endogenous α-globin gene (Figure S6A). Non-transduced ATM donor cells had an α/β-globin ratio of 0.0, while ratios for HD cells ranged from 0.88 to 2.50. On average, one copy of the integrated *HBA2* transgene resulted in ∼14% α-globin mRNA expression per β-globin mRNA for EV-α-UV, whereas EV-α achieved ∼30% (Figure 6D).

**Table 1:** Clinical presentation and ɑ/β-globin ratios by alpha thalassemia genotype

Genotype	Clinical presentation^21^	ɑ/β-globin mRNA median (range)^2^	ɑ/β-globin protein median (range)^22^
Normal (ɑɑ/ɑɑ)	normal state	1.3 (0.9–2.9)	1.04 (0.88–1.28)
Silent carrier (**ɑ−/ɑɑ**)	asymptomatic, hemoglobin and mean corpuscular volume (MCV) values may be near lower limits of normal	0.7 (0.3–1.2)	0.83 (0.72–1.05)
Alpha thalassemia trait (ɑ**−**/ɑ**−**) or (**−−**/ɑɑ)	asymptomatic, microcytotic anemia	0.5 (0.2–0.9)	0.69 (0.55–0.82)
HbH Disease (deletional) (ɑ−/−−)	mild to moderate anemia (hemoglobin [Hgb] = 10–12 g/dL in men and 9.0–10.5 g/dL in women), transfusions are rare	0.2 (0.1–0.2)	0.43 (0.34–0.55)
Alpha thalassemia major (ATM) (−−/−−)	hydrops fetalis (severe anemia, lethal without adequate pre-natal care); post-natal patients are fully transfusion dependent at birth	0 (0.0–0.0)	0 (0.0–0.0)

HPLC analysis of BM-derived RBCs showed that both EV-α and EV-α-UV restored α-globin with α/β-globin ratios ranging from 0.45 to 1.10, reaching HD levels (0.54–0.85) (Figure 6E). Moreover, β-globin dimers were reduced to less than 10% upon *HBA2* transgene expression (Figure S6B). In CB- and FL-derived RBCs, the ratios of α- to β- + γ_1_- + γ_2_- chains were restored, with values ranging from 0.17 to 0.40, compared with HD of 0.49 (Figure 6F). Additionally, α-globin expression levels exceeded 40% of endogenous levels in every donor and 100% of endogenous levels in BM-derived patient cells (Figure S6C).

HbA and HbF in ATM BM-derived RBCs reached 62%–94% of detected hemoglobin peaks (Figures 6G; Figure S6D). Normalizing HbA and HbF to VCN, EV-α and EV-α-UV yielded averages of 35% and 15%, respectively, comparable to one endogenous copy of *HBA2* (Figure S6E).

Single-cell RNA sequencing (RNA-seq) analysis was conducted in differentiated erythroid cells from the ATM BM-derived patient cells (EV-α VCN = 2 and EV-α-UV VCN = 4). The *HBA2* transgene was restored in erythroid cells, closely resembling the expression and distribution of the *HBA2* gene in cells from the two HDs (Figure 6H). The RNA expression was also displayed by a heatmap representing the top differentially expressed genes when comparing HDs with ATM samples. Such analysis reveals near-complete *HBA2* restoration, with levels matching those measured in HDs. Additionally, *TRIB3*, an important gene for erythroid maturation, was the second highest differentially expressed gene (excluding *HBA1*, *HBQ1*, and *HBQ2* that are part of the α-globin locus deletion in the patient and would not be restored with α-globin LV transduction) and showed partial restoration (Figures 6I and S6F). Increased expression of genes such as *LIZ*, *VIM*, *and GCNT1* in treated ATM cells suggested an improvement in erythroid homeostasis. Gene set enrichment analysis further revealed enrichment of the iron-ion binding pathway in HD cells, with treated ATM cells also showing upregulation in genes critical for iron homeostasis and heme synthesis (Figure S6G). Together, these findings emphasize the ability of EV-α and EV-α-UV to achieve high and near-complete *HBA2* restoration at the single-cell level, while also partially rescuing pathways essential for RBC function.

## Discussion

Once lethal *in utero* or shortly after birth, ATM is now compatible with survival and excellent neurologic outcomes due to advances in prenatal care and the use of IUTs.^3^^,^^23^ Although ATM is most prevalent in Southeast Asia, the incidence in the United States is rising due to migration patterns, making it an emerging clinical concern. Despite supportive interventions such as IUTs, chronic blood transfusions, and chelation therapy, there remains an urgent need for curative approaches. Several strategies are being explored, including genome editing and the upregulation of embryonic zeta-globin, to provide definitive therapeutic options.^24^

Inspired by the success of β-globin gene addition therapies, we developed a strategy to target α-thalassemia through LV-mediated α-globin gene addition HSC therapy. Partial globin expression is sufficient to alleviate disease severity in both β-and α-thalassemia. Notably, most patients with α-thalassemia with an α/β-globin ratio >0.2 are transfusion-independent and exhibit minimal symptoms.^2^ Therefore, our goal was to restore α-globin expression and rebalance the α/β-globin ratio in erythroid cells, thereby enabling the production of functional RBCs and restoring hemoglobin levels.

We designed short- to medium-sized α-globin EVs, containing the *HBA2* gene and various α- and β-globin regulatory elements. The top-performing vectors, EV-α and EV-α-UV, contain the full-length, non-codon-optimized *HBA2* gene, under the regulation of the core β-globin promoter and β-globin LCR. The integration of the β-globin LCR enhancer in every vector, rather than the α-globin HS40 enhancer, reflects its well-established and superior performance. Decades of clinical and pre-clinical data support the β-globin LCR as a robust, erythroid-specific, position-dependent regulatory element.^7^ In contrast, the α-globin HS40 enhancer is a much smaller and weaker enhancer and does not confer chromatin-opening capabilities, resulting in integration-dependent and potentially inconsistent transgene expression.^25^ The EV-α vector, with a longer β-LCR element, expressed higher levels of α-globin per vector copy, in the range of one endogenous *HBA* allele, while the shorter EV-α-UV vector transduced CD34^+^ cells more efficiently. This trade-off—higher expression per vector copy with longer LCR elements but reduced titer—has been observed previously.^12^^,^^14^

In HD CD34^+^ HSPCs, we observed high transgene expression in erythroid cells, with no detectable expression in myeloid cells, supporting the erythroid-specific profile of the α-globin EVs. Studies in mobilized peripheral blood stem cells (mPBSCs) demonstrated that EV-α achieved clinically relevant VCNs and produced α∗/β-globin mRNA ratio corresponding to levels observed in non-transfusion-dependent α-thalassemia patients and healthy individuals.^2^

*In vitro* toxicology studies indicated no adverse effects on mPBSCs transduced to high VCNs for survival, proliferation, or differentiation into myeloid or erythroid lineages. Both vectors demonstrated safety and efficacy in HSPCs in a xenograft model, achieving optimal gene transfer efficiency and narrow VCN distributions within the therapeutic range. This narrow distribution of VCN is critical in gene therapy as it minimizes the risks of insertional oncogenesis by reducing the likelihood of excessive vector integration near proto-oncogenes. Consistent with this safety profile, vector integration site analysis of xenografted human cells revealed safe, polyclonal integration patterns with high diversity and no enrichment near proto-oncogenes.

EV-α and EV-α-UV effectively reversed the α-thalassemia phenotype in HSPCs from three patients with ATM. Both vectors restored the α/β-globin chain ratios and hemoglobin levels to near or within HD ranges in differentiated RBCs. EV-α-UV achieved near-complete transduction and restored α-globin expression to HD levels in BM-derived patient RBCs, whereas EV-α maintained high α-globin expression with a clinically safe VCN of ≤5. Both vectors exceeded 50% of endogenous α-globin expression across all patient sources, reaching 100% in BM-derived RBCs. Transgene expression from both vectors reversed the ATM phenotype by reducing toxic β-globin dimers and HbH tetramers. Functional restoration was further supported by single-cell RNA-seq, showing nearly all transduced erythroid cells expressed the *HBA2* transgene.

### Conclusions

EV-α and EV-α-UV demonstrated high transduction rates, robust lineage-specific expression, and a favorable safety profile. Transduction with equivalent concentrations of EV-α and EV-α-UV vectors produced similar net α-globin levels, with EV-α compensating for its lower transduction and VCN with higher expression per vector copy. Restoration of α-globin chain production and hemoglobin levels in ATM patient-derived HSPC sources shows compelling therapeutic potential and strongly supports advancing into preclinical studies. Whereas EV-α-UV demonstrated higher viral titers, improved CD34^+^ cell infectivity, and more efficient gene transfer, EV-α produced higher α-globin expression per integrated vector copy, a critical factor for therapeutic efficacy. Additionally, EV-α maintained a favorable safety profile and efficient transduction of hematopoietic stem cells. Taken together, these data supported the selection of EV-α as the lead therapeutic candidate for ongoing preclinical studies. EV-α LV is shorter than all β-globin LVs currently used for sickle cell disease or β-thalassemia, and expresses sufficient levels of α-globin to improve erythropoiesis and efficiently transduce a high percentage of HSPCs, which should reduce the fraction of unprotected erythroid cells that undergo ineffective erythropoiesis. Ongoing work includes preparation for an investigational new drug application to initiate human trials to address the unmet medical needs of patients with ATM and ultimately extend therapeutic benefit to non-ATM α-thalassemia patients who are transfusion dependent, as seen in alpha thalassemia intermedia (hemoglobin H disease). Collectively, these efforts aim to deliver a beneficial gene therapy for α-thalassemia.

### Limitations of the study

While our study demonstrates vector safety in animal models and robust correction of α-globin expression in alpha thalassemia major donor cells, we did not evaluate *in vivo* disease correction in α-thalassemia animal models. Furthermore, although our integration site analyses demonstrated a polyclonal profile with no evidence of clonal expansion at four months post-transplant, longer-term studies with multiple time points will be necessary to assess clonal stability. These ongoing efforts are critical for advancing this approach toward clinical application and supporting our preparations for future human clinical trials.

## Resource availability

### Lead contact

Further information and requests for resources and reagents should be directed to and will be fulfilled by the lead contact, Dr. Donald B. Kohn (dkohn1@mednet.ucla.edu).

### Materials availability

EV-α and EV-α-UV lentiviral vectors, and all other vectors described here, may be obtained from the lead contact and under material transfer agreement from the University of California Regents. These vectors are the subject of a patent application filed by the University of California Regents. This study did not generate new unique reagents.

### Data and code availability

• Single-cell RNA-seq data have been deposited at GEO, accession number: GEO: GSE292575, and are publicly available as of the date of publication. For the vector integration site analysis, DNA library was sequenced using Illumina method, and genomic sequence alignments were mapped using human genome draft hg38. Integration site datasets were further aligned and bioinformatically analyzed using INSPIIRED pipeline (available at http://github.com/BushmanLab/INSPIIRED). All integration site DNA sequences are deposited at the NCBI short read with BioProject ID/accession number: PRJNA1225241.^19^^,^^20^
• Vector integration site analysis: DNA library was sequenced using Illumina method, and genomic sequence alignments were mapped using human genome draft hg38. Integration sites datasets were further aligned and bioinformatically analyzed using INSPIIRED pipeline (available at http://github.com/BushmanLab/INSPIIRED). All integration site DNA sequences are deposited at the NCBI short read with BioProject ID/accession number: PRJNA1225241.^19^^,^^20^
• This paper does not report original code.
• Any additional information required to reanalyze the data reported in this work paper is available from the lead contact upon request.

## Acknowledgments

E.E.R.S. would like to acknowledge and thank the following funding sources that enabled this work: 10.13039/100000050National Heart, Lung, and Bood Institute, National Institues of Health (RO1-HL161291, T.C.M.), 10.13039/100000900California Institute for Regenerative Medicine (CIRM) – (DISC2-13405, D.B.K.), and Broad Stem Cell Research Center (BSCRC) Fellowship by Rose Hills Foundation (UCLA, E.E.R.S.). E.E.R.S. also acknowledges funding for A.G.G. from the 10.13039/100000900California Institute for Regenerative Medicine through the Bridges to Stem Cell Research and Therapy Award (EDUC2-12718), which supported her nine-month collaboration on this project. T.C.M. was funded by the 10.13039/100000900CIRM CLIN2-09183, 10.13039/100000002NIH R01 161291, and funds from the 10.13039/100008069UCSF Center for Maternal-Fetal Precision Medicine.

The Flow Cytometry Core of the Eli & Edythe Broad Center for Regenerative Medicine and Stem Cell Research at UCLA provided technical support for this study. We extend our gratitude to the UCLA Technology Center for Genomics and Bioinformatics for conducting the 10× single-cell sequencing and to Eric Soupene at UCSF for performing the single globin chain HPLC analysis. We thank the VMHDS core at the University of Pennsylvania, RROD: SCR_022433, for their assistance with integration site analysis. Special thanks to Billie Lianoglou for her assistance with patient sample collection. We acknowledge that the graphical abstract and other panels throughout the main figures were created with biorender.com.

## Author contributions

E.E.R.S. executed all experiments and wrote the manuscript. E.E.R.S. and D.B.K. conceived and designed the experiments. K.H. contributed to the design and the execution of NSG assays. B.C.F. conducted the hemoglobin HPLC assays. K.T. assisted with the packaging and titering of lentiviral vectors. C.M., K.L., E.B., and E.S., as UCLA undergraduate students during this study period, contributed to portions of the experimental execution. A.G.G. supported the downstream analysis of ATM and NSG assays. K.M. and D.B.K. contributed to writing, editing the manuscript, and creating figures. T.C.M. provided cells from patients with ATM cells and provided guidance throughout the project. R.S. and D.S. conducted the single-cell RNA-seq analysis who are team members of M. K.C laboratory. F.B., J.E., and M.H. performed the vector integration site analysis. E.E.R.S. and D.B.K. are inventors on a patent application related to this work.

## Declaration of interests

E.E.R.S. and D.B.K. are inventors on a patent application covering the lentiviral vectors designed and developed in this study.

## STAR★Methods

### Key resources table

**Table:** 

REAGENT or RESOURCE	SOURCE	IDENTIFIER
**Antibodies**		

hCD45 (V450)	BD HorizonCAT #560367	RRID: AB_1645573
hCD34 (APC)	BD PharmingenCAT #555824	RRID: AB_398614
hCD34 (APC)	BioLegend CAT #343509	RRID: AB_1877154
hCD19 (Pe-Cy7)	BioLegendCAT # 302216	RRID: AB_314246
hCD56 (PE)	BD PharmingenCAT #555516	RRID: AB_395906
hCD3 (APC-Cy7)	BioLegendCAT #300318;	RRID: AB_314053
hCD33 (BV711)	BioLegendCAT #303424	RRID: AB_2565775

**Bacterial and virus strains**		

NEB® Stable Competent *E*. *coli* (High Efficiency)	New England BioLabs	C3040I

**Biological samples**		

Mobilized CD34^+^ Stem/Progenitor Cells; GCSF; 1 × 10e6 cells, Cryo	Charles River Laboratories	N/A
Human Bone Marrow Mononuclear Cells, Frozen	STEMCELL	CAT #70001.2
Human Cord Blood Mononuclear Cells, Frozen	STEMCELL	CAT #70007.1
Human Fetal Liver Mononuclear Cells, Frozen	UCLA	N/A

**Critical commercial assays**		

PureLink™ Genomic DNA Mini Kit	ThermoFisher	K182001
RNeasy Kits for RNA Purification	Qiagen	74104
QIAGEN Plasmid Kits for Plasmid DNA Extraction	Qiagen	12162
SuperScript™ III First-Strand Synthesis System	ThermoFisher	18080051

**Deposited data**		

RNA-seq	For reviewers: GSE292575: Go to https://www.ncbi.nlm.nih.gov/geo/query/acc.cgi?acc=GSE292575 Enter token ipkfemkwtnwnvev into the box.	Accession number: GEO: GSE292575
Vector Integration Site	VMHDS core, University of Pennsylvania http://github.com/BushmanLab/INSPIIRED	RRID:SCR_022433^20^ accession number: PRJNA1225241^19^^,^^20^

**Experimental models: Cell lines**		

HUDEP-2	https://doi.org/10.1371/journal.pone.0059890	N/A

**Experimental models: Organisms/strains**		

NSG mice (NOD.Cg-Prkdc^scid^ Il2rg^tm1Wjl^/SzJ)	Jackson Laboratories	Cat# 005557

**Software and algorithms**		

SnapGene	SnapGene	7.2.1
GraphPad Prism	GraphPad	10.4.1
Loupe Browser	Loupe Browser	8.0.0
R software	www.r_project.org	R.4.4.1

### Experimental model and study participant details

#### HUDEP-2 cells

HUDEP-2 human erythroid progenitor cells with and without the homozygous 20 kb deletion encompassing the α-globin loci were provided by the MacKenzie laboratory (UCSF). As an immortalized cell line, sex characteristics are not applicable, and the influence of sex or gender on study results was not assessed. HUDEP-2 cells were thawed in IMDM supplemented with 20% fetal bovine serum and freshly added 1× glutamine, penicillin, streptomycin. Cells were maintained at a concentration of 3E5 cells per mL in StemSpan SFEM medium, supplemented with 1 μM dexamethasone and 1% glutamine, penicillin, streptomycin. Medium was supplemented with 1 μg/μL doxycycline, 50 ng/uL human stem cell factor.

#### Human CD34^+^ hematopoietic stem and progenitors cells (HSPCs)

CD34^+^ HSPCs were obtained from both healthy donors (HDs) and patients with alpha thalassemia major (ATM). CD34^+^ cells from healthy donor mobilized peripheral blood (mPB), bone marrow (BM), and fetal liver (FL) were purchased from STEMCELL Technologies as pre-enriched CD34^+^ populations. These cells were collected under protocols approved by the respective institutional review boards (IRBs), and informed consent was obtained from all donors. Because the cells were purchased fully anonymized, no additional ethics approval was required for this study.

ATM patient samples were collected under UCSF IRB-approved protocols (IRB #10–00350 and #16–21157). Specifically, BM CD34^+^ cells were isolated from a 1-year-old patient with a homozygous SEA deletion (20 kb *HBA2/1*), who received intrauterine and chronic postnatal transfusions. The patient participated in a clinical trial that entailed a bone marrow sample taken at one year of age and extra material used for these studies; CB CD34^+^ cells were from a fetus with intrauterine transfusions; and FL CD34^+^ cells were obtained from a 17-week fetus diagnosed by prenatal testing. The influence of sex or gender on experimental outcomes was not evaluated.

CD34^+^ HSPCs were plated overnight (1E5-1E6 cells/mL) in a 96-well plate pre-coated with RetroNectin. Cells were cultured in 100uL X-VIVO 15 medium, supplemented with glutamine, penicillin, streptomycin and human stem cell factor, human Flt-3 ligand, human thrombopoietin, and human IL-3.

#### Animal studies

All animal procedures were performed under UCLA Institutional Animal Care and Use Committee (IACUC)–approved protocols. NOD. Cg-Prkdcˆscid Il2rgˆtm1Wjl/SzJ (NSG) mice were purchased from Jackson Laboratories (Cat# 005557) and housed in specialized barrier facilities for immunocompromised mice, with a maximum of four mice per cage. Eight-week-old mice of mixed sex were used for transplantation experiments. Mice underwent cytoreduction via 250 cGy Cesium-137 irradiation at least three days after arrival, followed by retro-orbital transplantation of human CD34^+^ HSPCs. Animals were euthanized 16 weeks post-transplantation for downstream analyses. No sex-dependent differences were analyzed in this study.

### Method details

#### Cloning and vector production

One series of α-globin lentiviral erythroid vectors (α-globin EVs) were designed based on the GLOBE LV vector, a clinically validated β-globin SIN lentiviral vector utilized in clinical trials for β-hemoglobinopathies.^9^^,^^10^ The UV-based α-globin vector series were based on the UV LV, optimized for shorter regulatory elements.^12^^,^^26^ To introduce the desired elements of the series of α-globin EVs, the *HBB* gene was excised from the plasmid backbones of GLOBE and UV using reverse-oriented primers, and the genomic 835-bp *HBA2* gene sequence (GRCh38/hg38; chr16:172,876-173,710) was cloned using the NEBuilder HiFi DNA Assembly kit (New England Biolabs, Ipswich, MA, USA, Cat# E2621S). Each additional element, such as the core 256bp core region of HS40 (MSC-R2) (chr16:113,500-113,755), the 210bp core alpha promoter (chr16:172,666-172,875), and the *HBA2* cDNA, were integrated similarly. All plasmids were verified by Sanger sequencing (Laragen, Culver City, CA, USA, and Primordium Labs, Los Angeles, CA, USA). Vectors tested in human primary cells contained an 18-nucleotide addition (tcgccgcagagcagcaca) (TAG) downstream of the *HBA2* translational STOP codon.

Lentiviral vector particles were produced via transient transfection of a HEK 293T cells with knockout of Protein Kinase RNAse (PKR) using the third-generation packaging system and adding using sodium butyrate (Sigma Aldrich, St. Louis, MO, USA, Cat# 303410).^16^^,^^27^ Raw viral supernatants were harvested at 72 h and titered using HT-29 cells. For vector assessment in primary cells, viral vector supernatants were concentrated ∼230× by a 90-min ultracentrifuge spin (26,000 × g) and resuspended in X-VIVO 15 (Lonza, Basel, Switzerland, Cat# BEBP04-744Q).^28^ Functional titers (transducing units/mL or TU/mL) were determined by droplet digital PCR (ddPCR, Bio-Rad, Hercules, CA, USA, QX200), measuring vector copy numbers at various dilutions.

#### HUDEP-2 Cell culture and differentiation

The HUDEP-2 α-globin knockout cells were provided by MacKenzie laboratory. CRISPR-Ca9 with two guides were utilized to create the homozygous 20kb deletion encompassing the four α-globin loci (guides: AGGTTCTAGCCCCTGAGCAC and CCTCCAAGTAACTGGGACAT). HUDEP-2 cells were thawed in IMDM (Thermo Fisher Scientific, Waltham, MA, USA, Cat# 12440061) supplemented with 20% fetal bovine serum (FBS) (Omega Scientific, Tarzana, CA, USA, Cat# FB-02) and freshly added 1× glutamine, penicillin, streptomycin (PSG) (Gibco, Grand Island, NY, USA, Lot #2587132). Cells were maintained at a concentration of 3E5 cells per mL in StemSpan SFEM medium (STEMCELL Technologies, Canada, Cat# 09655) supplemented with 1 μM dexamethasone (DEX) (Sigma Aldrich, St. Louis, MO, USA, Cat# D2915) and 1% PSG. Medium was supplemented with 1 μg/μL doxycycline (DOX) (Sigma Aldrich, Cat# D9891), 50 ng/uL human stem cell factor (hSCF) (Thermo Fisher Scientific, Cat#300-07).

Differentiation was performed over a period of 10 days post transduction. Cells were plated at 2.5E5 cells/mL in 500 μL IMDM supplemented with 2% FBS (Omega Scientific), 3% human AB serum (Sigma Aldrich, Cat# H4522), 10 μg/mL insulin (Sigma Aldrich, Cat# I9278), 3 IU/mL heparin (Hikma Pharmaceuticals), and 1% PSG. For the first 6 days, cells were maintained at a concentration of 3E5 cells/mL and media was supplemented with 200 μg/μL holo-transferrin (Sigma Aldrich, Cat# 11096-37-0), 1 μg/μL DOX, 30 ng/mL, erythropoietin (EPO) (Jassen Pharmaceuticals), 10 ng/uL hSCF (Thermo Fisher) and 1 ng/mL IL-3 (Thermo Fisher Scientific, Cat#200-03). On days 7–12, cells were maintained at a concertation of 5E5 cells/mL and media was supplemented with 500 μg/mL holo-transferrin, 1 μg/mL DOX, and 30 ng/mL EPO. Cells were analyzed for VCN on day 7, and hemoglobin by HPLC on Day 12.

#### Isolation of CD34^+^ HSPCs

CD34^+^ HSPCs from healthy and ATM donors were isolated using microbeads-conjugated anti-human CD34 antibodies (Miltenyi Biotec, Bergisch Gladbach, Germany, Cat# 130-046-702). The CD34 purity of isolated cells was assessed via flow cytometry using CD34-APC antibody (BioLegend, San Diego, CA, USA, Cat# 343509). HD mPBSC, BM or FL HSPC were purchased as CD34-enriched cells (Stemcell Technologies, Vancouver, BC, Canada).

#### ATM patient cells

Cells were obtained from three patients with ATM at UCSF, each homozygous for a 20 kb *HBA2/1* SEA deletion, under appropriate IRB protocols (IRB:10–00350, and IRB: 16–21157). BM CD34^+^ cells came from a 1-year-old patient who had intrauterine transfusions and chronic postnatal transfusions. CB CD34^+^ cells were from a patient with intrauterine transfusions. FL CD34^+^ cells were obtained from a 17-week fetus diagnosed via prenatal testing.

#### *In vitro* erythroid differentiation

CD34^+^ HSPCs were plated overnight (1E5-1E6 cells/mL) in a 96-well plate pre-coated with RetroNectin (TakaraBio, Cat.#T100B). Cells were cultured in 100uL X-VIVO 15 medium (Lonza), supplemented with glutamine, penicillin, streptomycin (PSG) (Gibco, Grand Island, NY, USA, Lot #2587132), and human stem cell factor (hSCF) (Thermo Fisher Scientific, Cat#300-07), human Flt-3 ligand (Thermo Fischer, Cat#300-19), human thrombopoietin (hTPO) (Thermo Fischer, Cat #300-18), and human IL-3 (Thermo Fisher Scientific, Cat#200-03), as previously detailed by Romero et al.^29^ Transduction was performed on the day after plating (day 1) using concentrated vector particles at different concentrations, ranging from 1E5 - 2E7 TU/mL.

Erythroid differentiation was conducted over 18–21 days. Differentiation medium contained Iscove’s Modified Dulbecco’s Medium (IMDM) (Life Technologies), 1% PSG, 330 μg/mL of holo-transferrin (Sigma Aldrich) (reconstituted with Na_2_HPO_4_, NaH_2_PO_4_, NaCl, and water), 10 μg/mL recombinant human insulin (Sigma-Aldrich), 2 IU/mL heparin (Hikma Pharmaceuticals), and 5% inactivated human plasma (Sigma-Aldrich). On days 2–7, cells were cultured at a density ranging from 5E5-2E6 cells/mL and supplemented with 1uM hydrocortisone (Sigma-Aldrich), 5 ng/mL IL-3 (Thermo Fischer), 100ng/mL hSCF (Thermo Fischer), and 3 U/mL of erythropoietin (EPO). Cells were then transferred to flasks containing a pre-formed adherent stromal cell layer (MS-5, murine stromal cell line, provided by Gay Crooks, UCLA), and cultured with EPO until days 11 and without EPO until days 18–21.

#### VCN and mRNA quantification by droplet digital PCR (ddPCR)

On day 14 of erythroid differentiation, 5E5 cells were collected for genomic DNA extraction, and 2.5E5 cells were collected for RNA extraction to quantify VCN and α- and β-globin mRNA, respectively. Genomic DNA and RNA were isolated using the PureLink Genomic DNA (MiniKit (Invitrogen, Carlsbad, CA, USA, Cat# K182001) and RNeasy Plus Mini Kit (QIAGEN, Hilden, Germany, Cat# 79254). To eliminate DNA contamination, RNA samples underwent DNase digestion using RNase-Free DNase Set (QIAGEN) prior to the first wash during purification. The purified RNA was subsequently reverse-transcribed into cDNA, as previously described (Thermo Fisher Scientific, Waltham, MA, USA, RNaseOUT Recombinant Ribonuclease Inhibitor: Cat# 10777019, Random Primers: Cat# 48190011, M-MLV Reverse Transcriptase: Cat#: 28025013).^29^

For VCN quantification, ∼50ng of genomic DNA (or 1.1uL) was added to each 20uL ddPCR reaction, followed by droplet generation and PCR amplification, as outlined by Hindson et al.^30^ VCN values were determined using primer and probe sets targeting the vector’s HIV-1 PSI sequence (primers: AAGTAGTGTGTGCCCGTCTG and CCTCTGGTTTCCCTTTCGCT, probe: CCCTCAGACCCTTTTAGTCAGTGTGGAAAATCTCTAG) and normalized to a reference human gene, human Syndecan 4 (SCD4), that was amplified as described by Urbinati et a (primers: CAGGGTCTGGGAGCCAAGT and GCACAGTGCTGGACATTGACA, probe: CCCACCGAACCCAAGAAACTAGAGGAGAAT).^31^ To note, HUDEP-2 cells have a VCN of 2, and therefore 2 was subtracted in all transduced HUDEP-2 cells.

To measure total α-globin and transgene-specific α∗-globin mRNA levels, two sets of primers and probes were designed. One set detected the transcripts for both the endogenous α-globin and the transgene-derived “tagged” α-globin transcripts (endogenous primers: cttccccaccaccaagac and taggagcttgaagttgaccg, probe: cgcacttcgacctgagccacg) The second set specifically targeted the unique “TAG” region introduced during cloning, located between the STOP codon and the 3′UTR of the *HBA2* transgene mRNA, allowing precises quantification of α-globin transgene expression. β-globin mRNA levels were quantified using a separate set of primers and probe specific to β-globin transcripts (transcript specific primers: TCAACTTCAAGCTCCTAAGCC and ctctgcggcgaTTAACGGTA, probe: TGCCTGCTGGTGACCCTGGC). The quantification of both α-globin and β-globin mRNA enabled the evaluation of the α/β-globin mRNA ratio (β-globin primers: GGA GAA GTC TGC CGT TAC TG and CAC TAA AGG CAC CGA GCA CT, probe: AA CCT CTG GG TCC AAG GGT AGA CCA CCA GCA G).

#### Hemoglobin and single globin chain analysis and quantification via HPLC

On day 14 or days 18–21 of *in vitro* erythroid differentiation, 3E6 cells were collected for hemoglobin tetramer analysis and single globin chain quantification. Cells were centrifuged at 500 × g for 5 min, and the resulting red pellets were vigorously vortexed and lysed overnight in Hemolysate reagent (2E5/μL) (Helena Laboratories). The red cell lysates were collected the following day after centrifugation at 5152 × g for 5 min at 4°C and stored at −80°C until further analysis.

Tetrameric hemoglobin protein complexes, including HbF and HbA, were characterized by reverse-phase HPLC (HPLC, Infinity 1260, Agilent) using a weak cation-exchange PolyCAT A, PolyLC column.^31^ HbA and HbF were identified by comparing to known elution times of standards, and the relative percentages were calculated based on the area under the curve of other peaks found on the chromatograms, analyzed using OpenLAB CDS Chemstation software.

For single globin chain quantification, cell pellets were frozen and shipped on dry ice to the HPLC Core at University of California San Francisco for analysis. Reverse-phase HPLC was performed using a Vydac 214 TP C4 column with a mobile phase of 10% methanol in acetonitrile and elution with 0.5% trifluoroacetic acid adjusted at a pH 2.9. Quantification of globin chains was conducted using TotalChrom software (PerkinElmer Flexar), and the resulting data were exported to GraphPad Prism for visualization and analysis.

#### Mice

All animal experiments were conducted in compliance with protocols by the UCLA Animal Research Committee under the Division of Laboratory of Medicine. NSG mice (NOD.Cg-Prkdc^scid^ Il2rg^tm1Wjl^/SzJ) were purchased from Jackson Laboratories and housed in a specialized barrier facility designed for immunocompromised mice, with no more than four mice per cage (Jackson Laboratories, Bar Harbor, ME, USA, Cat# 005557). At 8 weeks of age, at least three days after arrival, mice underwent bone marrow cytoreduction via a single dose of 250 centi-gray from a Cesium-137 irradiator. HSPC transplantation was performed via retro-orbital injection of 100uL PBS (Corning, Corning, NY, USA, Cat# 21-040-CV) -containing the transplanted cells. Sixteen weeks post-transplantation, mice were euthanized and bones from the hind legs were harvested. The harvested bones were crushed to isolate human CD45^+^ cells, using human isolation kit (Miltenyi Biotec, Bergisch Gladbach, Germany, Lot #130-052-301).

#### Colony Forming Unit (CFU) assay

BM CD34^+^ HSPCs (100 per replicate) were plated in MethoCult (StemCell Technologies, Cat# H4435), on the day of bone marrow harvest from the NSG mice (16 weeks post-transplant). Cultures were maintained at 37°C with 5% CO_2_ for 14 days, after which mature colonies were counted and scored under a microscope. Myeloid and erythroid colonies were enumerated separately.

For further analysis, individual myeloid colonies were picked and transferred to 96-well plate containing PBS to dissolve the methylcellulose by overnight incubation. Plates were centrifuged at 2,000 × g for 5 min, and the supernatant was removed. The resulting pellets were resuspended in 25uL of Quick Extract Buffer (Biosearch Technologies, Novato, CA, USA, Cat# QE09050) and incubated at 60°C for an additional 10 min. The extracted DNA (∼4uL) was subsequently used for VCN analysis of individual progenitor colonies.

#### Vector integration site analysis

Genomic DNA was extracted from purified human CD45^+^ cells isolated the bone marrow of NSG mice 16 weeks post-transplantation. Vector integration site analysis (VISA) was conducted at the VMHDS core at the University of Pennsylvania, RRID:SCR_022433.^20^ DNA samples were sheared by sonication, followed by adaptor ligation and two-rounds of ligation-mediated PCR, resulting in the attachment of specific linkers at each end of DNA fragments. DNA library was then sequenced using Illumina method, and genomic sequence alignments were mapped using human genome draft hg38. Integration sites datasets were further aligned and bioinformatically analyzed using INSPIIRED pipeline (available at http://github.com/BushmanLab/INSPIIRED). All integration site DNA sequences are deposited at the NCBI short read with BioProject ID/accession number: PRJNA1225241.^19^^,^^20^

#### Single Cell RNA-sequencing (scRNA-seq) and analysis

On day 16 of *in vitro* erythroid differentiation, 120,000 cells were collected and resuspended in 100uL of Phosphate Buffered Saline (PBS) (Corning) with 0.05% Bovine Serum Albumin (BSA) (Sigma Aldrich, Cat# A7979-12) for single cell RNA sequencing conducted by the UCLA Technology Center for Genomics and Bioinformatics. Single-cell libraries were constructed using the Chromium Next GEM Single Cell 3′ Kit v.3.1 (10× Genomics) targeting 10k cells, following manufacturer’s protocol. The libraries were sequenced on the Illumina NovaSeq X+ paired-end platform (on a 10B flow cell with 150 + 10+24 + 150 cycles) and data de-multiplexing was performed using BCLConvert software v4.3.13 (Illumina). Sequenced reads in FASTQ format were aligned and analyzed via the CellRanger Count pipeline v7.2.0 (10× Genomics) using the gex-GRCh38-2020A (10× Genomics) reference database under default settings.

Single-cell RNA sequencing (scRNA-Seq) of *in vitro* differentiated red blood cells (RBCs) was performed to ensure robust quality control, sample demultiplexing, barcode processing, alignment, and single-cell 3′ gene counting. Further QA/QC steps were conducted using the *Partek Flow* software, v12.3. Gene and feature annotation was performed using hg38_ensembl_release90_v2. Genes detected in fewer than 10 cells or cells expressing fewer than 100 genes were excluded to minimize noise in downstream analyses. Normalization of individual samples was performed using cell RNA content, as implemented by the cell ranger count pipeline. For comparative analyses, only genes detected in at least ten cells were included. Further, counts per million (CPM) normalization was applied to all samples using *Partek Flow v12*.*3*. Downstream differential analyses were performed using *Partek Flow v12*.*3* software for cell clustering, gene expression and pathway analysis. Principal Component Analysis (PCA) was applied to the normalized, log-transformed, centered, and scaled gene-barcode matrix to reduce dimensionality. Data were visualized in a two-dimensional space using t-distributed Stochastic Neighbor Embedding (t-SNE). Cell clustering was based on their PCA space projections using both graph-based and k-means clustering methods. Cluster classification and annotation were performed based on known marker genes for distinct erythroid lineage stages (e.g., GYPA, HBB, ALAS2, and other relevant cell-specific markers). Differentially expressed genes (DEGs) in specific erythroid lineages were identified relative to all other clusters using ANOVA. Gene set enrichment analysis on sample-wise DEG comparisons was performed using Gene Ontology enrichment analysis and visualization tool (GOrilla) wherein the enrichment scores were plotted in a heatmap. Individual gene expression data were shown in forms of tSNE gradient (low to high) and violin dot plots at the single-cell level.

### Quantification and statistical analysis

A Wilcox test was used to measure the statistical difference in the VISA study found in Table S1. In Figure 5, analysis of NSG assay was based on ANOVA (analysis of variance) across all arms with selected statistics shown, with *p* < 0.05 considered significant. Non-significance (ns) was set at *p* < 0.05. Error bars for all represent the mean with standard deviation, unless otherwise noted.

## Supplemental information

Document S1. Figures S1–S6 and Tables S1 and S2

Document S2. Article plus supplemental information
