# Supplementary material for: Lentiviral vectors for hematopoietic stem cell gene therapy restore α-globin expression in α-thalassemia red blood cells
Source: Cell Rep Med. 2025 Sep 17;6(10):102362. doi: 10.1016/j.xcrm.2025.102362 (PMC12629793; doi:10.1016/j.xcrm.2025.102362)
Supplement: Document S1. Figures S1–S6 and Tables S1 and S2 [file mmc1.pdf]

Cell Reports Medicine, Volume *6*

Supplemental information

Lentiviral vectors for hematopoietic stem cell gene therapy restore α-globin expression in α-thalassemia red blood cells

Eva E.R. Segura, Kevyn Hart, Beatriz Campo Fernandez, Devin Brown, Kevin Tam, Andrea Gutierrez Garcia, Eva Seigneurbieux, Karen Li, Carol Mulumba, Emma Blakely, Katelyn Masiuk, Roshani Sinha, Devesh Sharma, John Everett, Matthew Hogenauer, M. Kyle Cromer, Frederic Bushman, Tippi C. MacKenzie, and Donald B. Kohn

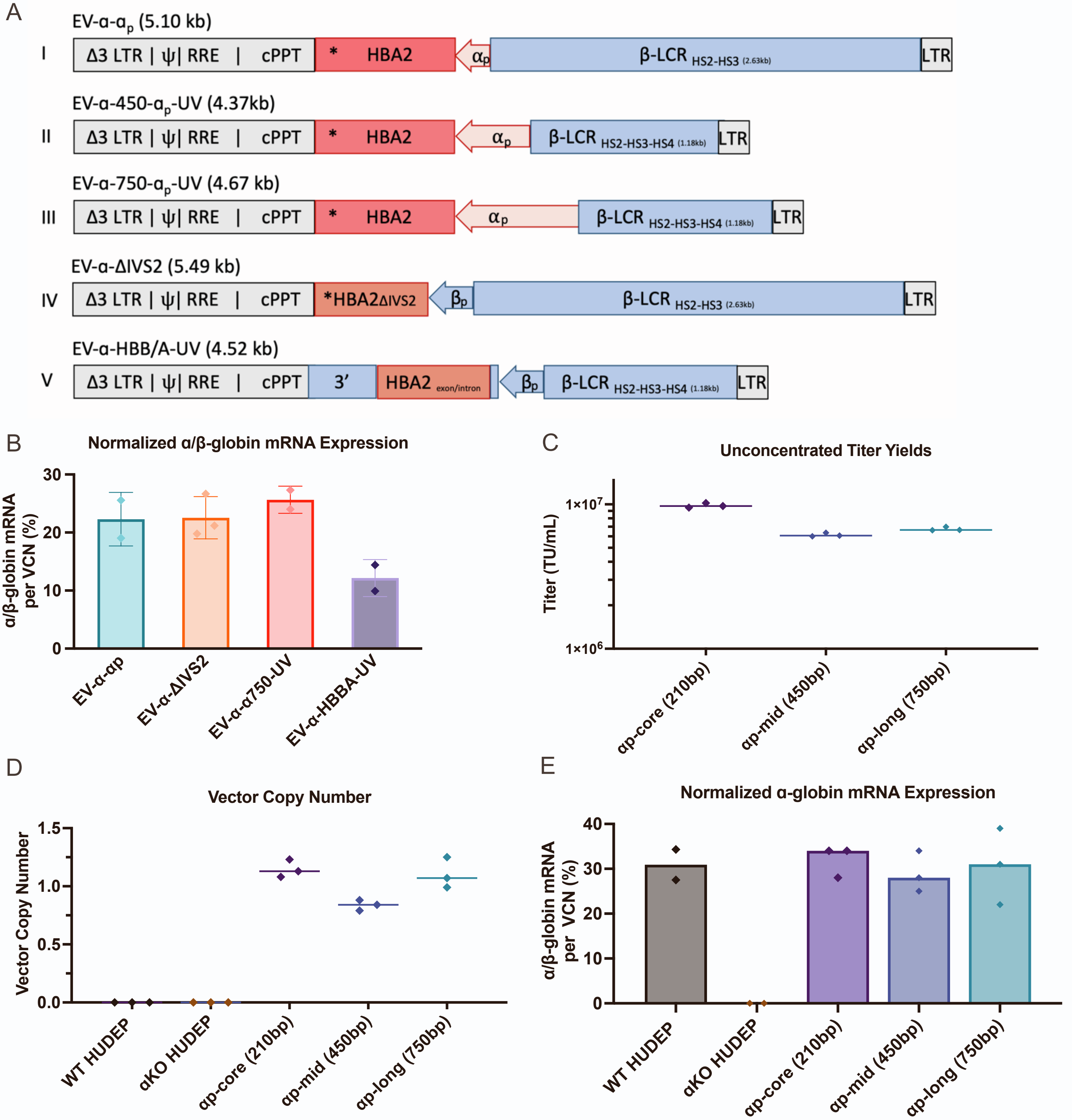

Supplemental Figure 1: Additional design and assessment of α-thal EVs in α-globin knockout (α-KO) human erythroid cell line (HUDEP).

A) Schematic representation of α-globin erythroid vectors (α-globin EVs) in their proviral forms. Viral elements are shown in gray: LTR: Long Terminal Repeat with a 400 bp deletion in the HIV U3 region (Δ3); packaging signal (φ or PSI), Rev Responsive Element (RRE), central polypurine tract (cPPT). β-globin elements are in blue: β-globin core promoter (βp, 265 bp), β-globin Locus Control Region (β-LCR) comprising combinations DNase I hypersensitive sites (HS2, HS3 and HS4; 1.2–2.6kb), and the β-globin 5' and 3' UTR. α-globin elements are shown in red: (835 bp, including 3' and 5' UTRs and all introns), and the α-globin core promoter (α_p, 210, 450, 750 bp). EV-α-IVS2 lacks intron 2. The *HBA2* gene is in reverse orientation relative to vector transcription to prevent intron splicing. **B)** Experimental set-up: α-globin KO cells were transduced with EV-α at 5E5 TU/mL and cultured in erythroid differentiation conditions from day 2 to day 5. On day 5, cells were harvested for genomic DNA (gDNA) for the assessment of VCN and RNA for α-globin and β-globin expression. Gene expression analysis of transgene-derived α- and endogenous β-globin mRNA showing the α*/β-globin ratio, normalized to corresponding to VCN. **C)** Small-scale samples of each vector without concentration were produced in triplicates in HT-29 cells and their determined titer as determined in Materials and Methods. **D-E)** Following in vitro erythroid differentiation in α-KO HUDEP cells **D:** VCN in α-KO HUDEP cells, **E:** Gene expression analysis of transgene-derived α- and endogenous β-globin mRNA showing the α/β-globin ratio, normalized to corresponding to VCN. Error bars represent mean with standard deviation (SD).

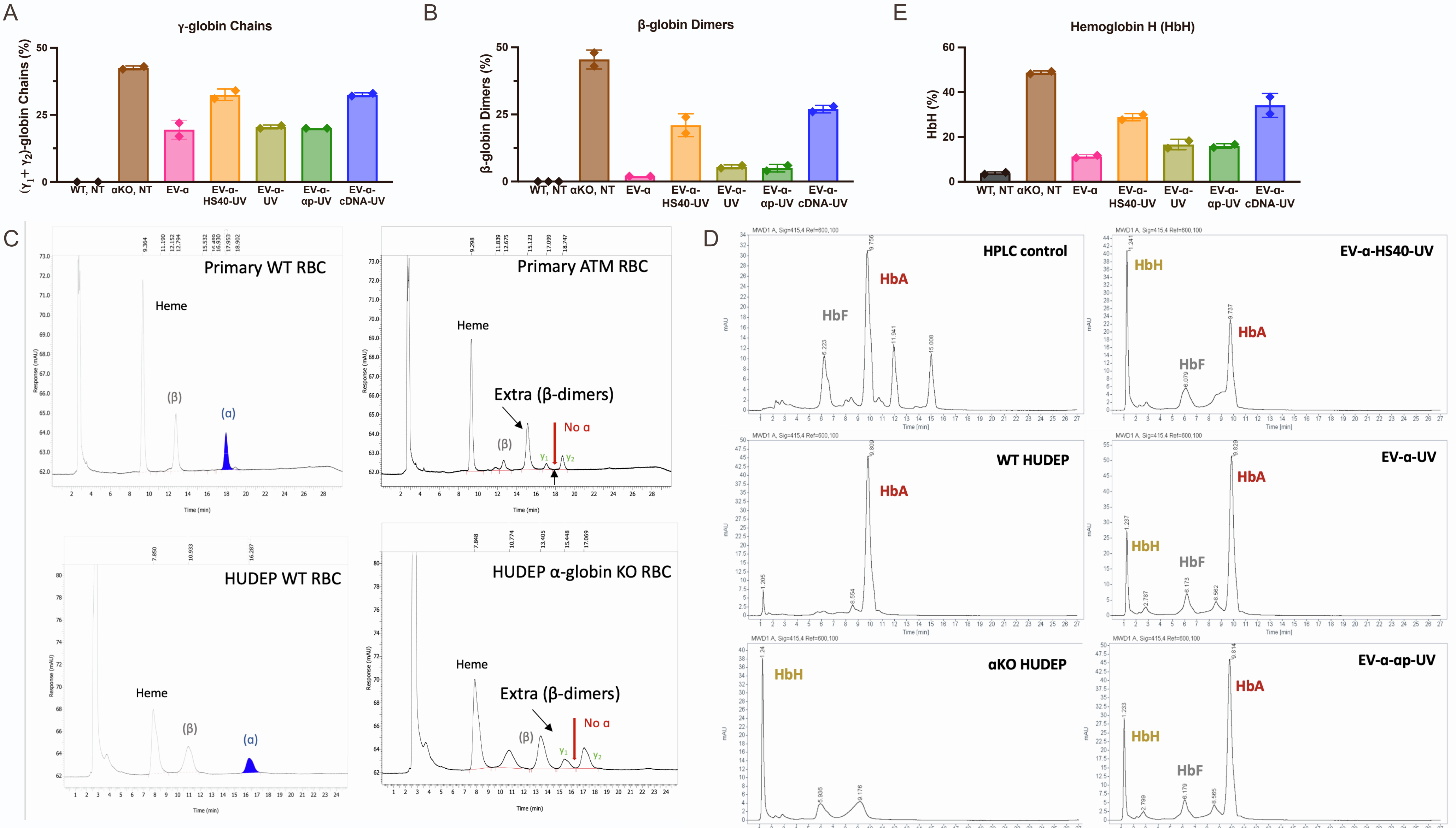

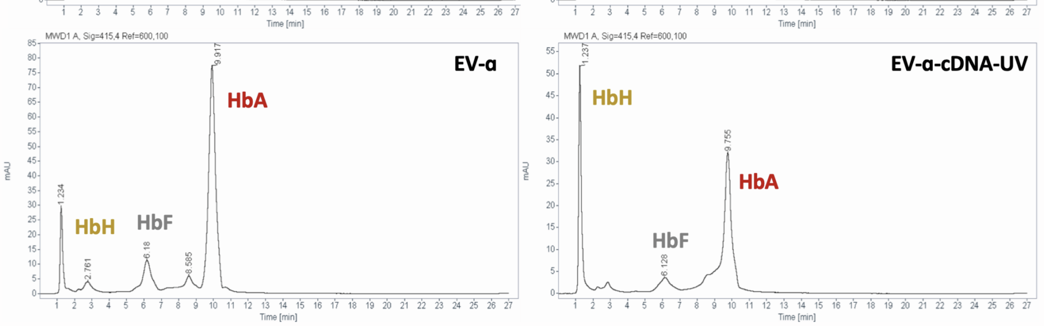

Supplemental Figure 2: Protein analysis of α-thal EVs in α-KO HUDEP.

In vitro erythroid differentiation in α-KO HUDEP cells for 12 days. Quantification and HPLC chromatograms of single globin chain or hemoglobin tetramers **A)** Percentage of γ-globin chains (*HBG1* and *HBG2)* **B)** Percentage of β-globin dimers. **C)** Single globin HPLC chromatograms of erythroid differentiated WT HUDEP-2, α-KO HUDEP-2 cells, healthy and alpha thalassemia major donors (HD and ATM, respectively). The α-globin peak is present in WT or HD donors (blue) and absent in α-globin KO HUDEP-2 and ATM donor cells. The two minor peaks, found in α-globin KO HUDEP-2 and ATM cells, are γ-globin peaks. **D)** Representative HPLC chromatograms of hemoglobin tetramers. Control chromatograms indicate standardized peaks for the hemoglobin column: Fetal hemoglobin (HbF) elutes as ~6 minutes, adult hemoglobin (HbA) elutes at ~10 minutes, and β-globin tetramer (HbH, β_4,) elutes at ~1.2 minutes in α-KO HUDEP cells. **E)** Percentage of hemoglobin H (β-globin tetramers, β_4). Error bars represent mean with standard deviation.

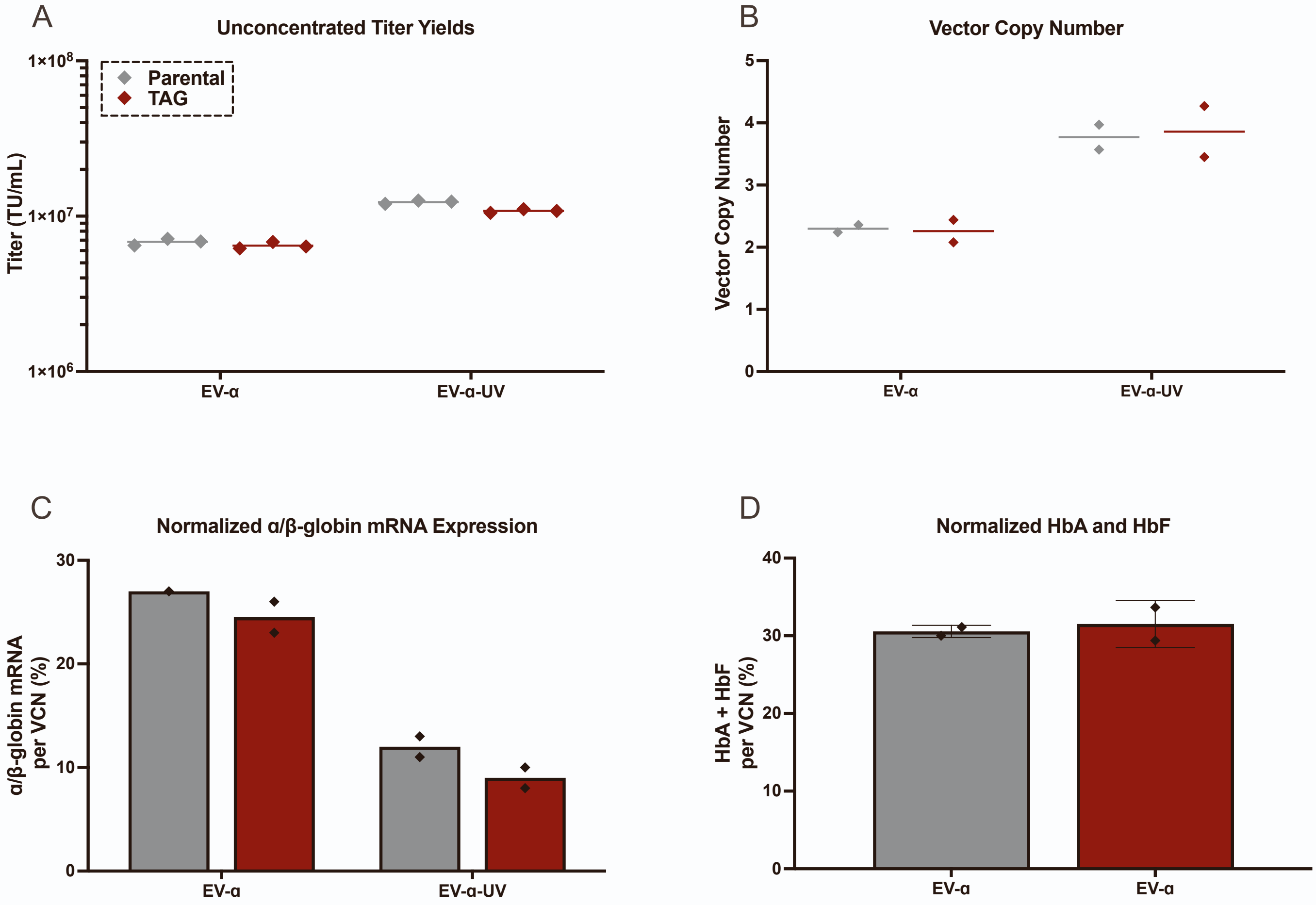

Supplemental Figure 3: Comparative vector efficiency of tagged versus parental α-thal EVs

A) Small-scale samples of EV-α without concentration were produced in triplicates in HT-29 cells and their determined titer as determined in Method Detail. **B-D)** Experimental set-up: α-globin KO cells were transduced with EV-α or EV-α-UV at 5E5 TU/mL and cultured in erythroid differentiation conditions from day 2 to day 5. On day 5, cells were harvested for genomic DNA (gDNA) for the assessment of VCN and RNA for α-globin and β-globin expression. Cells were further differentiated to mature RBCs and analyzed on day 12 via HPLC for single globin chain and tetramer hemoglobin production**. B:** VCN in α-KO HUDEP cells. **C:** Gene expression analysis of transgene-derived α- and endogenous β- globin mRNA showing the α/β-globin ratio, normalized to corresponding to VCN. **D:** Relative percentages of HbA and HbF, calculated as the area under their respective HPLC peaks relative to the total HPLC area, normalized to corresponding VCN. Bars and error bars represent mean with standard deviation.

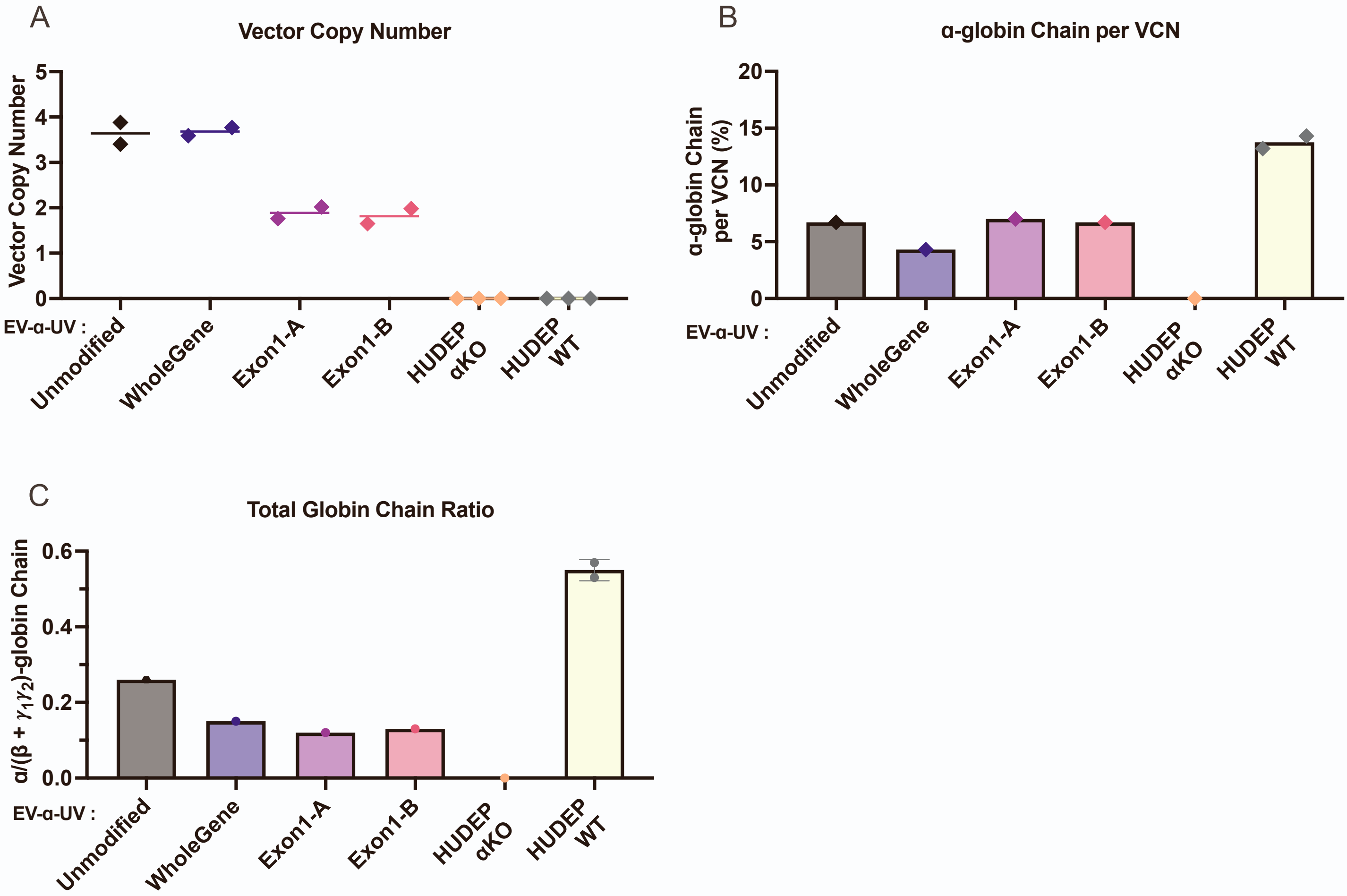

Supplemental Figure 4: Assessment of codon-optimized EV-α-UV

Experimental set-up: α-globin KO cells were transduced with α-globin EVs at 5E5 TU/mL and cultured in erythroid differentiation conditions from day 2 to day 5. On day 5, cells were harvested for genomic DNA (gDNA) for the assessment of VCN and analyzed on day 12 via HPLC for single globin chain production. **A)** VCN in α-KO HUDEP cells. **B)** Normalization of α-globin chain production per VCN. **C)** Normalized α*/(β+y)-globin ratio per VCN, or per one *HBA2* endogenous copy in WT HUDEP cells (total WT expression divided by 4, representing the 4 *HBA2* and *HBA1*).

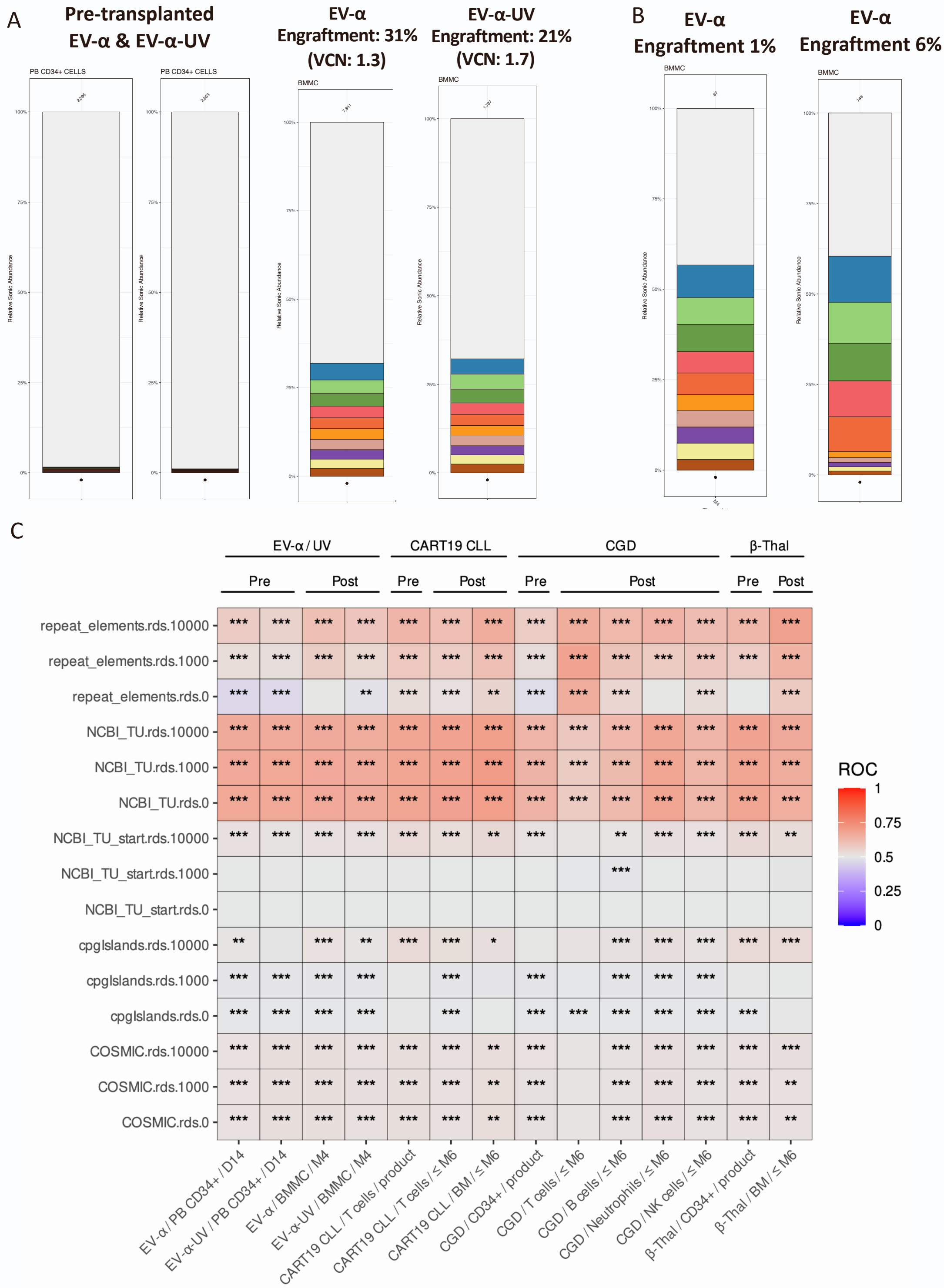

Supplemental Figure 5: VISA-based clonal and genomic characterization of EV-α or EV-α-UV integration profiles. Vector integration site analysis (VISA), performed by ligation-mediated PCR and next generation sequencing, and aligned to the human genome. A) Relative abundance of the top 10 most abundant clones in pre-transplanted and of engrafted cells transduced with EV-α or EV-α-UV. B) Top 10 most abundant clones in samples with lowest engraftment profile. C) Comparison of lentiviral vector integration site distributions between cells transduced with the LV EV-α vector and distributions from lentiviral-vector based clinical trials where integration-mediated adverse events were not reported. Each column indicates a different clinical trial; gene therapies targeted cancer by lentiviral generation of CART cells [1]; CGD by treatment of hematopoietic stem cells [2]; and beta-thalassemia also by treatment of hematopoietic stem cells [3]. For each of these trials, no integration-mediated adverse events were reported. Data in each column as either pre-infusion products (Pre) or post-transfusion samples (Post). Rows indicate genomic features mapped on the human genome (draft hg38). The oncogene annotation is from the Cosmic oncogene list v10. The integration site frequencies are compared to random distributions using the ROC area method; the scale on the right indicates enrichment (red) or depletion (blue) relative to random [4]. Because the size of genomic windows most relevant for comparisons are not known in advance, three different window sizes were compared per feature (0 = within the feature; 1,000 = within the feature or within 1,000 bases; 10,000 = within the feature or within 10,000 bases). P-values for comparison to random are indicated on each tile in the heat map as follows: * = p-value <0.05, ** = p-value <0.01, *** = p-value < 0.001. As can be seen in the figure, no major differences were seen between the EV-α vectors and the LV trials in which adverse events were not reported.

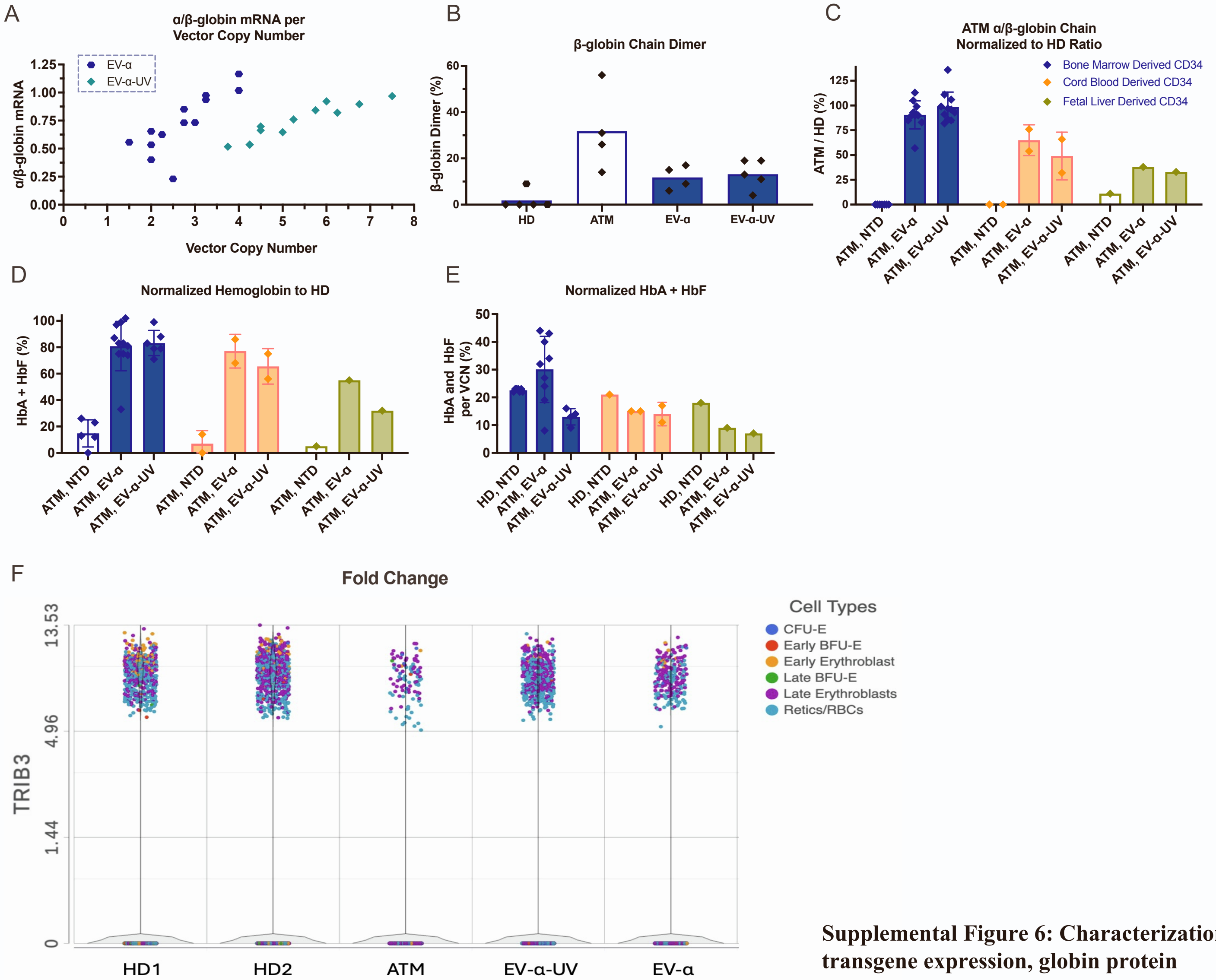

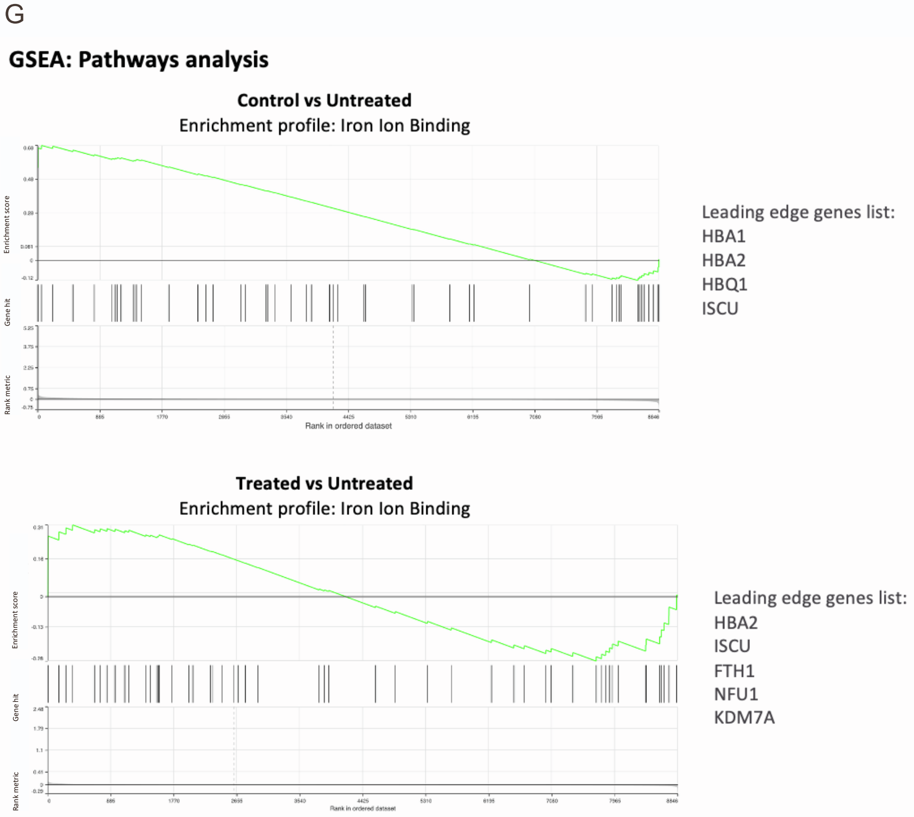

Supplemental Figure 6: Characterization of transgene expression, globin protein restoration and transcriptomic correction in alpha thalassemia major (ATM) donor red blood cells

Experimental setup for transduction, with EV-α or EV-α-UV, and differentiation of BM, CB and FL CD34+ HSPCs. Cells were analyzed for VCN, mRNA expression, and protein production via HPLC on days 14 and 21. (BM: bleu, CB: orange, FL: green). A) Expression of α-globin HBA2 transgene from EV-α and EV-α-UV at different VCNs. B) β-globin chain dimers. C) α/β-globin chain ratio normalized to levels in HD cells. D) HbA and HbF production normalized to levels in HD RBCs. E) HbA and HbF production normalized to VCN. Error bars represent mean with standard deviation. BM cells analyzed from single cell RNA-sequencing. F-G) Single-cell RNA-SEQ analysis was conducted in differentiated erythroid cells from the ATM BM-derived patient cells. F: Dot plots of fold change of gene expression of TRIB3, erythroid maturation marker. G) Gene Set Enrichment Analysis (GSEA) of iron-ion binding pathway. HD cells compared to ATM patient cells (top) and treated ATM cells compared to non-treated ATM patient cells (bottom).

Supplemental Table 1. Summary of in vivo vector performance and clonal diversity in the VISA study

Mouse	Vector	TU/ mL	VC N	hCD45	Inferred cells #	Unique Integrants	Gini	Shannon Indices	>20% clones	Highest Gene	In or Near Proto-Oncogene
1	EV-α	6E6	1.3	31	7381	1001	0.80	5.04	Negative	CD2AP	No
2	EV-α	6E6	2.5	1	67	34	0.34	3.32	Negative	DEPTOR	No
3	EV-α	2E7	1.4	48	1029	317	0.59	4.95	Negative	RBM4	No
4	EV-α	2E7	1.3	13	691	382	0.40	5.32	Negative	SMARCA4	No
5	EV-α	2E7	2.4	6	746	250	0.64	3.96	Negative	RERE	No
6	EV-α	2E7	3.0	31	3053	418	0.78	4.55	Negative	CD84	No
1	EV-α-UV	6E6	2.3	74	8143	499	0.866	4.22	Negative	NCOR1	No
2	EV-α-UV	2E7	3.4	48	6028	803	0.725	5.43	Negative	DDX29	No
3	EV-α-UV	2E7	2.9	63	21355	2405	0.809	6.1	Negative	FOXN3	No
4	EV-α-UV	2E7	1.7	21	1737	421	0.685	4.85	Negative	SPATS2	No
5	EV-α-UV	2E7	3.5	22	1756	297	0.779	3.78	Negative	ABCA6	1.50%
6	EV-α-UV	2E7	3.5	30	19563	3549	0.771	6.33	Negative	PDSSB	0.04%

VCN: Vector Copy Number. Transduction measured in TU/mL. Engraftment is measured in % of human CD45+ cells (hCD45) . The Gini coefficient measures the inequality in clonal abundance within each sample. A coefficient of zero indicates equal abundance across all sites (polyclonal), while higher values suggest increasing dominance of fewer clones (oligoclonal). The Shannon index is a metric for diversity, considering both the abundance and evenness of integration events. A threshold of >20% clones is the clinical limit triggering further safety analysis. When comparing the two vectors, no statistical differences were observed. A Wilcox was performed, yielding p values greater than 0.05. The significant values Gini index: 0.476, Shannon index: 0.914, number of integrated sites (nSites), 0.352 (not shown), and number of integrated cells (Inferred Cells): 0.171.

Supplemental Table 2. Demographics and cell sources of ATM donors

**Patient/Donor Genotype**	**Cell Source**	**Race/Ethnicity**	**Age**
SEA/SEA*	Bone Marrow (aspirate)	Vietnamese, Chinese and Laotian	1 year
SEA/SEA	Cord Blood	Filipino	0 day
SEA/SEA	Fetal Liver	Chinese and Vietnamese	16-week gestation

*Alpha Thalassemia Major (ATM) with the SEA deletion refers to the Southeast Asian mutation caused by homozygous 20kb deletion encompassing both the *HBA2* and *HBA1* genes.

1. Nobles CL, Sherrill-Mix S, Everett JK, et al. CD19-targeting CAR T cell immunotherapy outcomes correlate with genomic modification by vector integration. *Journal of Clinical Investigation*. 2019;130(2):673-685. doi:10.1172/JCI130144

2. the Net4CGD consortium, Kohn DB, Booth C, et al. Lentiviral gene therapy for X-linked chronic granulomatous disease. *Nat Med*. 2020;26(2):200-206. doi:10.1038/s41591-019-0735-5

3. Boulad F, Maggio A, Wang X, et al. Lentiviral globin gene therapy with reduced-intensity conditioning in adults with β-thalassemia: a phase 1 trial. *Nat Med*. 2022;28(1):63-70. doi:10.1038/s41591-021-01554-9

4. Berry CC, Nobles C, Six E, et al. INSPIIRED: Quantification and Visualization Tools for Analyzing Integration Site Distributions. *Molecular Therapy - Methods & Clinical Development*. 2017;4:17-26. doi:10.1016/j.omtm.2016.11.003
